# Preoperative CT deep learning of tumor and visceral adipose tissue combined with postoperative pathology predicts colorectal cancer recurrence: a multicenter study

**DOI:** 10.3389/fmolb.2026.1937172

**Published:** 2026-09-24

**Authors:** Pengfei Wei, Linhua Zhong, Yongming Tan, Jinhong Zhao, Yongjie Zhou

**Affiliations:** 1 Department of Radiology, Jiangxi Cancer Hospital & Institute, Jiangxi Clinical Research Center for Cancer, The Second Affiliated Hospital of Nanchang Medical College, Nanchang, China; 2 Department of Radiology, The First Affiliated Hospital, Jiangxi Medical College, Nanchang University, Nanchang, China; 3 Department of Radiology, The Second Affiliated Hospital, Jiangxi Medical College, Nanchang University, Nanchang, China

**Keywords:** colorectal cancer, computed tomography, deep learning, recurrence-free survival, visceral adipose tissue

## Abstract

**Introduction:**

To develop and externally validate a preoperative CT imaging model integrating primary tumour and visceral adipose tissue (VAT) features for predicting recurrence in colorectal cancer (CRC).

**Materials and methods:**

This retrospective multicentre study included 1,369 patients with CRC undergoing upfront curative-intent resection, comprising a training cohort (n = 705) and two external test cohorts (n = 425 and 239). A 3D convolutional neural network extracted tumour-centred volumetric features, while an attention-based multiple instance learning branch aggregated whole-abdominal VAT slices into patient-level representations. The two CT-derived scores constituted the preoperative imaging model. A clinicopathological component was combined with the imaging scores to construct the postoperative multimodal model; robustness to variable selection was evaluated using an all-candidate ridge-penalised Cox sensitivity model.

**Results:**

Both tumour and VAT DL scores remained independently associated with recurrence-free survival after multivariable adjustment. In external test cohorts 1 and 2, the preoperative Tumor + VAT imaging model achieved C-indices of 0.734 and 0.772, whereas the postoperative multimodal model achieved C-indices of 0.772 and 0.810 and 2-year time-dependent AUCs of 0.757 and 0.836, respectively. Training-derived tertile cut-offs from the postoperative model consistently stratified patients into low-, intermediate- and high-risk groups across all cohorts (all log-rank P < 0.001). In the two external cohorts, the clinicopathological-only model achieved C-indices of 0.709 and 0.715, compared with 0.772 and 0.810 for the postoperative multimodal model.

**Conclusion:**

The preoperative CT model non-invasively stratified recurrence risk using tumour and whole-abdominal VAT phenotypes, while postoperative clinicopathological features further improved prognostic performance.

## Introduction

Colorectal cancer (CRC) remains a major cause of cancer-related mortality worldwide (Bray et al., 2024). Even after curative-intent resection and adjuvant therapy, postoperative recurrence or metastasis remains common, particularly within the first 3 years (van der Stok et al., 2017). Current postoperative risk stratification relies largely on TNM stage and conventional clinicopathologic factors (Dekker et al., 2019). However, patients within the same stage can have markedly different recurrence risks, reflecting substantial clinical and biological heterogeneity (Jiang et al., 2024). This heterogeneity underscores the need for more comprehensive tools to estimate recurrence-free survival (RFS) and guide personalised treatment and surveillance.

CRC recurrence is shaped by interactions between tumour-intrinsic biology and host factors. Visceral adipose tissue (VAT) is a metabolically active endocrine and immune organ that can promote tumour progression through adipokine release, inflammatory signalling and metabolic dysregulation (Khandekar et al., 2011; Chaplin et al., 2022; Wang et al., 2026). Because abdominal computed tomography (CT) is routinely acquired during CRC staging and treatment planning (Benson et al., 2024), it offers a non-invasive opportunity to capture both tumour morphology and VAT-related body-composition features.

Quantitative imaging studies have used handcrafted CT radiomics to predict recurrence and metastasis in CRC (Badic et al., 2022; Liu et al., 2021). However, radiomic workflows depend on predefined features and can be sensitive to segmentation, acquisition and feature-selection choices. Deep learning (DL) can learn hierarchical representations directly from imaging data, reducing reliance on manual feature engineering (LeCun et al., 2015). Existing DL models in CRC, however, are often tumour-centred and based on 2D inputs, which may discard volumetric information (Liu et al., 2021; Lu et al., 2021). Conversely, many body-composition studies simplify VAT assessment to a single CT slice, potentially missing spatial heterogeneity across the intra-abdominal fat compartment (Zhou et al., 2025a; Zhou et al., 2026). Combining tumour features that reflect intrinsic aggressiveness with whole-abdominal VAT features that capture host metabolic-inflammatory status may therefore provide complementary prognostic information. Advanced DL architectures are well suited to this task. Three-dimensional convolutional neural networks (CNNs) are designed to model volumetric medical images and can preserve three-dimensional spatial context within the primary lesion (Soomro et al., 2019), whereas attention-based multiple instance learning (MIL) can aggregate slice-level VAT representations into a patient-level embedding when only patient-level outcome labels are available (Ilse et al., 2018; Chikontwe et al., 2021). This design allows the model to identify informative VAT slices without manual slice-level annotation.

Here, we developed and externally validated a preoperative CT imaging model integrating primary tumour and whole-abdominal VAT features. We then incorporated a seven-variable clinicopathological component to construct a postoperative multimodal model and quantify the incremental prognostic value of postoperative pathology. We also used activation mapping to visualise salient image regions and improve the interpretability of model predictions.

## Materials and methods

### Study patients

This retrospective multicentre study was approved by the Ethics Committee of Jiangxi Provincial Cancer Hospital (approval no. 2024ky071), which waived the requirement for written informed consent. All patient data were anonymised before analysis. Patients were enrolled from three medical centres. Patients treated at Centre 1 from July 2018 to June 2023 formed the training cohort, whereas patients treated at Centres 2 and 3 from January 2019 to September 2022 served as two independent external test cohorts. To prevent data leakage, the external test cohorts were excluded from model fitting, variable selection, feature standardisation, cut-off determination and hyperparameter tuning.

Eligible patients met the following criteria: (a) pathologically confirmed CRC after curative-intent resection; (b) no preoperative antitumour treatment; and (c) availability of a standardised preoperative contrast-enhanced abdominal CT scan acquired within 2 weeks before surgery. Of 1,523 patients assessed for eligibility, 31 were excluded because of another malignancy, 56 because of inadequate CT image quality or lesions too small for reliable detection and delineation, and 67 because of incomplete baseline clinicopathologic data. Ultimately, 1,369 patients were included: 705 in the training cohort, 425 in external test cohort 1 and 239 in external test cohort 2 (Figure 1).

**FIGURE 1:**
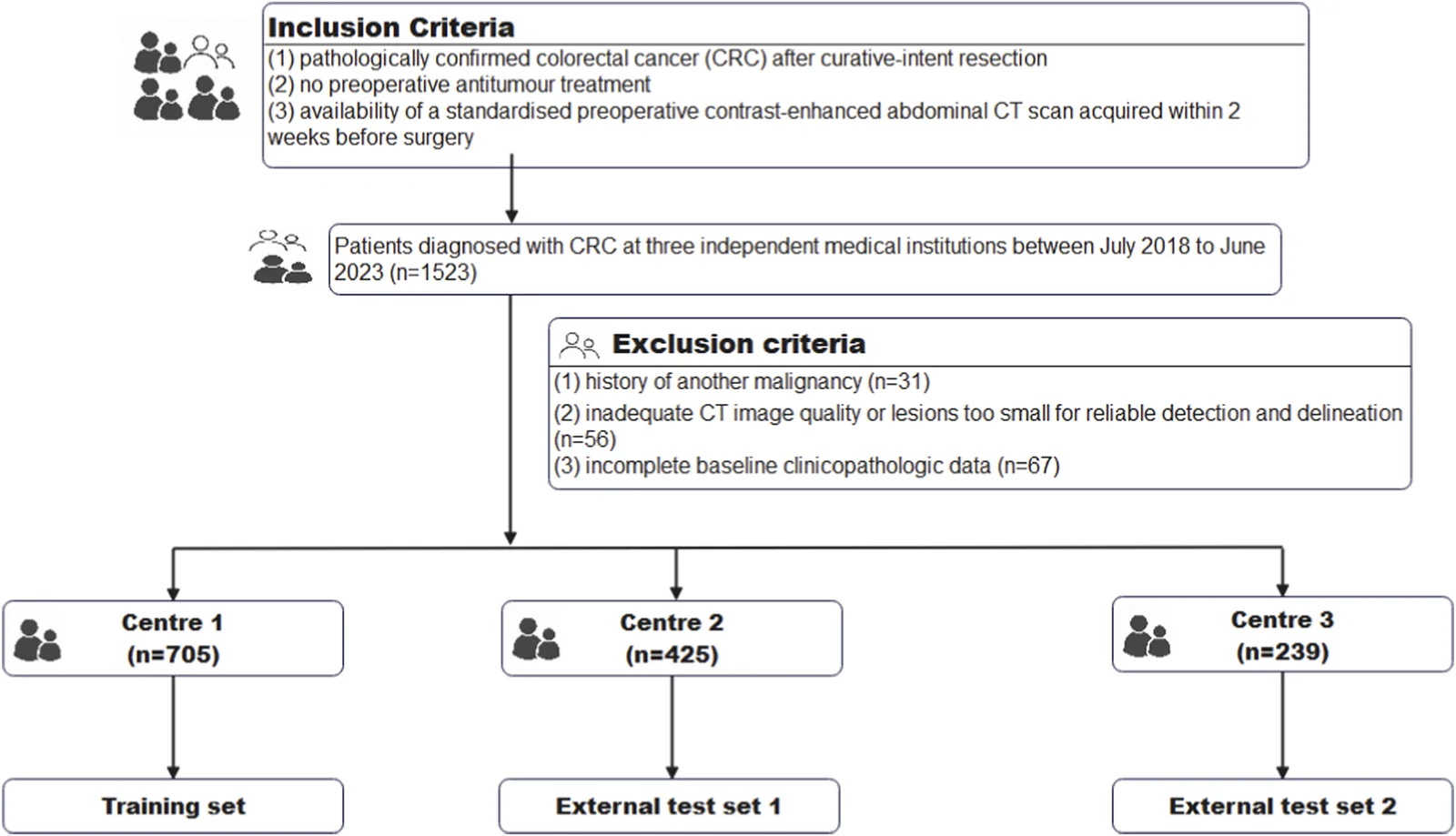
Patient selection and cohort allocation. Across the three participating centres, 1,523 patients were assessed for eligibility and 154 were excluded: 31 because of another malignancy, 56 because of inadequate CT image quality or lesions too small for reliable detection and delineation, and 67 because of incomplete baseline clinicopathologic data. The final study population comprised 1,369 patients: 705 from Centre 1 in the training cohort, 425 from Centre 2 in external test cohort 1 and 239 from Centre 3 in external test cohort 2. Historical retrospective screening logs retained exclusion reasons in aggregate; a reliable centre-specific breakdown of excluded patients could not be reconstructed.

### Follow-up and recurrence assessment

Follow-up commenced on the date of colorectal cancer resection, with assessments every 3 months during the first 2 postoperative years and every 6 months thereafter. Recurrence was confirmed by imaging, endoscopy, or pathological examination. RFS was defined as the interval from surgery to the first documented recurrence; patients without recurrence were censored at the last assessment confirming recurrence-free status. Missing subsequent visits were not interpreted as continued recurrence-free follow-up. First recurrences were classified as local, distant, or synchronous local and distant recurrence. Follow-up duration was estimated using the reverse Kaplan–Meier method and summarised by the median, interquartile range, observed range, and numbers at risk at 12, 24, and 36 months. Because visit-level attendance, external follow-up, and harmonised death data were unavailable, recurrence-free censoring before 24 months was conservatively considered potential early loss to follow-up, and death could not be modelled as a separate competing event. Further definitions and sensitivity analyses are provided in the Supplementary Material.

### CT image acquisition, segmentation and reproducibility

All patients underwent preoperative contrast-enhanced abdominal CT, and portal venous phase images were used for DL analysis. Scanner and reconstruction parameters are detailed in Supplementary Table S1. Primary tumours were manually delineated by an abdominal radiologist with 12 years of experience and reviewed by a senior radiologist with 35 years of experience, with discrepancies resolved by consensus. VAT was automatically segmented using TotalSegmentator version 2.13.0 with the tissue_4_types task and torso_fat output (Wasserthal et al., 2023). All segmentations underwent blinded visual quality control, and no voxel-level correction was applied to the VAT masks.

Tumour-segmentation reproducibility was assessed in 30 randomly selected patients using independent delineation by a second radiologist and repeated delineation by the original reader after 1 month. Mask agreement was evaluated using the Dice coefficient, Jaccard index and HD95. The locked tumour model was applied without retraining to assess the stability of Tumor DL scores across segmentations. Detailed procedures and results are provided in the Supplementary Material.

### Image preprocessing and deep learning model development

CT attenuation values were clipped to −200 to 300 HU and normalised to 0–1. Tumour volumes were cropped in native array space with a 12-voxel margin and resized to 80 × 80 × 80 voxels. For the VAT branch, 32 slices were deterministically sampled at evenly spaced quantiles between the 5th and 95th percentiles of VAT-containing slices and resized to 160 × 160 pixels. CT images and masks were interpolated using linear and nearest-neighbour methods, respectively. Both branches received two-channel inputs comprising masked CT and the corresponding binary mask. Identical preprocessing was applied across all cohorts; no slice duplication, scanner-specific harmonisation or post-cache augmentation was used. Two independently trained imaging branches were developed (Figure 2). The tumour branch used a residual 3D CNN to generate a continuous Tumor DL score, whereas the VAT branch combined a shared 2D CNN with attention-based multiple-instance learning to aggregate slice-level features into a patient-level VAT DL score (Ilse et al., 2018). Neither branch used transfer learning or explicit anatomical-coordinate encoding. Both branches were trained using a mini-batch approximation to the Cox partial-likelihood loss, enabling time-to-event supervision in the presence of censoring. Complete network architectures and mathematical definitions are provided in Supplementary Table S2.

**FIGURE 2:**
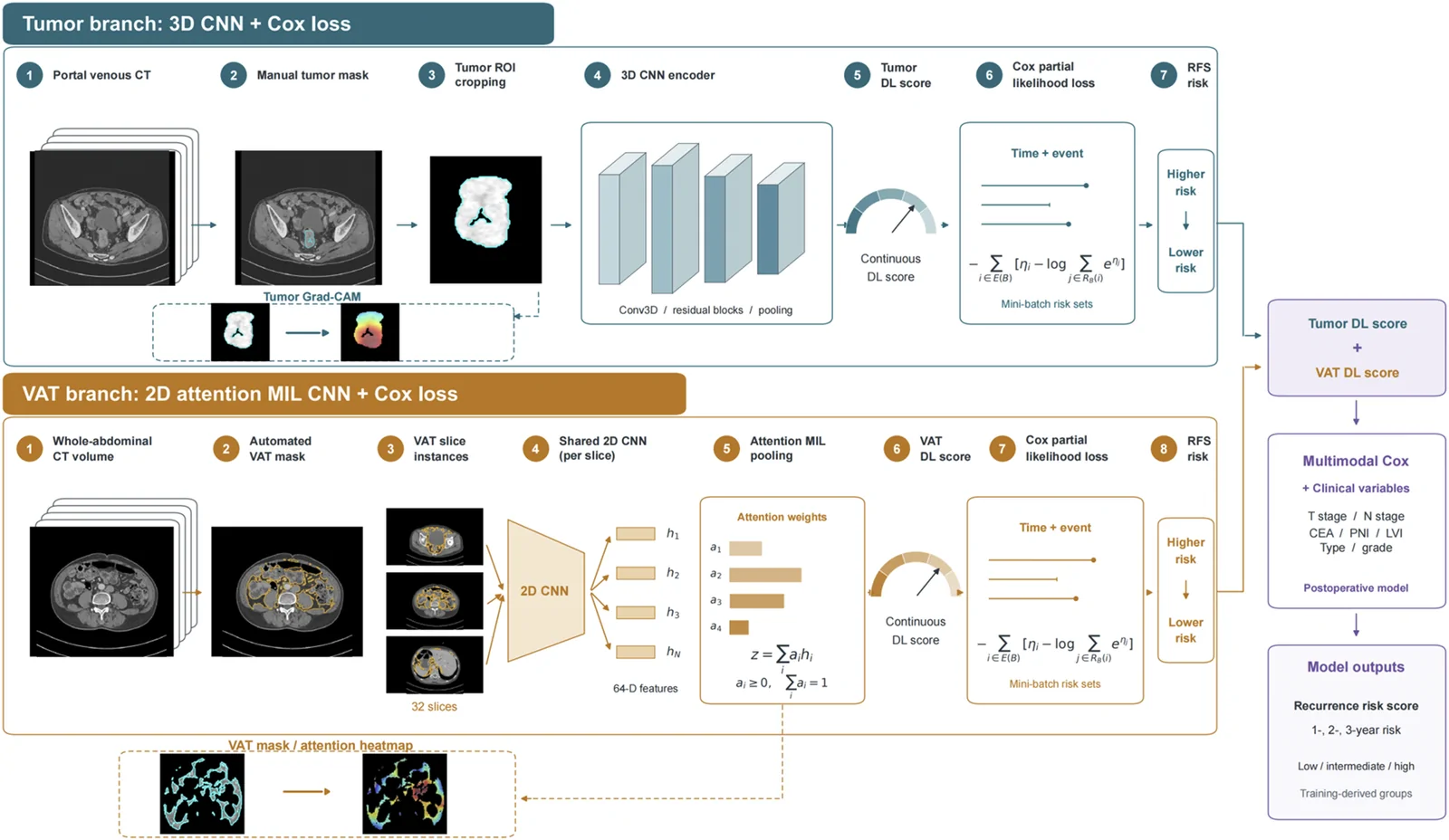
Workflow of the multimodal CT-based deep learning framework for recurrence-risk prediction. The tumour branch used portal-venous-phase CT images and manual tumour masks to crop tumour-centred regions of interest, extract volumetric features with a 3D convolutional neural network (CNN), and generate a tumour deep learning (DL) score under Cox partial-likelihood supervision. Tumour Grad-CAM was used to visualise image regions contributing to tumour-branch predictions. The visceral adipose tissue (VAT) branch used whole-abdominal CT volumes and automated VAT masks to form VAT-containing slice instances, extract slice-level features with a 2.5D CNN, aggregate them through attention-based multiple instance learning, and generate a VAT DL score. VAT attention heatmaps were used to localise informative adipose-tissue regions. Tumour and VAT DL scores were then combined with clinicopathologic variables in a multimodal Cox model to estimate recurrence risk.

The development cohort was stratified by recurrence status into an internal training subset of 564 patients (138 events) and a validation subset of 141 patients (35 events; seed 2026). Models were trained using AdamW and mixed precision, and checkpoints were selected according to internal-validation C-index. External cohorts were excluded from model selection, feature standardisation, hyperparameter tuning and cut-off determination. Internal stability was assessed using three repeats of five-fold cross-validation. Physical-space tumour preprocessing, positional encoding for VAT and complete-risk-set Cox estimation were examined in sensitivity analyses. Full preprocessing, training and sensitivity-analysis procedures are provided in the Supplementary Material.

### Preoperative imaging and postoperative multimodal risk modelling

Standardised Tumor and VAT DL scores were combined using Cox regression (Cox, 1972) to construct the preoperative Tumor + VAT imaging model. Twelve candidate clinicopathological variables were evaluated by univariable Cox analysis in the training cohort. Seven variables with P < 0.05 (CEA, N stage, pathological type, differentiation grade, perineural invasion (PNI), lymphovascular invasion (LVI) and dichotomised T stage [T1–2 vs. T3–4]) were included in the clinicopathological-only model and, together with both DL scores, the postoperative multimodal model. N stage was coded ordinally (N0 = 0, N1 = 1 and N2 = 2), with its hazard ratio representing a one-category increase. All coefficients were estimated in the training cohort and locked for external evaluation. Because the multimodal model incorporated postoperative histopathological variables, it was defined as a postoperative recurrence-risk model. An all-candidate ridge-penalised Cox model, with the penalty selected by repeated 10-fold cross-validation, was prespecified as a sensitivity analysis.

### Model evaluation and interpretation

Model discrimination was evaluated using Harrell’s C-index and cumulative/dynamic time-dependent area under the curve (AUC) at 12, 24 and 36 months. Prediction error was assessed using time-dependent Brier scores and the integrated Brier score (IBS). All models were locked before external evaluation and were not retrained or recalibrated. Gradient-weighted class activation mapping (Grad-CAM) was used to visualise regions contributing to deep learning risk scores (Selvaraju et al., 2017). Tumour heatmaps identified influential tumour-centred regions, whereas VAT attention weights and heatmaps indicated informative slices and adipose regions. These visualisations were interpreted qualitatively and not as evidence of biological causality. Detailed evaluation methods and case-selection criteria are provided in the Supplementary Material.

### Statistical analysis

Continuous variables were summarised as means with standard deviations or medians with interquartile ranges, and categorical variables as frequencies and percentages. Cohort comparisons used Student’s t-test or the Mann–Whitney U test for continuous variables and the chi-square or Fisher’s exact test for categorical variables. Survival associations were assessed using Cox regression. Model discrimination, calibration, prediction error and clinical net benefit were evaluated at 12, 24 and 36 months using methods accounting for censoring. Robustness was examined through informative-censoring, competing-risk, alternative CT-window, penalised-model, treatment-adjusted and subgroup analyses. Paired patient-level bootstrap resampling with 1,000 iterations was used for confidence intervals and model comparisons. All tests were two-sided, with P < 0.05 indicating statistical significance. Analyses were performed in Python, with deep learning models implemented in PyTorch. Full statistical procedures are provided in the Supplementary Methods. Reporting was informed by the TRIPOD + AI and CLAIM guidance (Collins et al., 2024; Tejani et al., 2024).

## Results

### Patient characteristics

Among 1,369 patients with colorectal cancer, recurrence occurred in 173 of 705 patients (24.5%) in the training cohort, 99 of 425 (23.3%) in external test cohort 1 and 60 of 239 (25.1%) in external test cohort 2. Baseline clinicopathological characteristics were broadly comparable across cohorts (Table 1), although standardised mean difference analyses identified greater heterogeneity in CT voxel spacing than in clinical or pathological variables. The locked final model remained robust across centres, tumour locations, sex and adjuvant-treatment groups, whereas its association with recurrence varied by pathological tumour-node-metastasis stage (Supplementary Table S3; Supplementary Figure S1).

**TABLE 1:** Baseline clinical characteristics of patients with colorectal cancer.

Characteristics	Training set (n = 705)	External test set 1 (n = 425)	External test set 2 (n = 239)	P value**
Age*	58.0 [51.0, 67.0]	58.0 [51.0, 67.0]	60.0 [50.0, 68.0]	0.370
Tumour location				0.992
Colon	374 (53.0%)	224 (52.7%)	127 (53.1%)	
Rectum	331 (47.0%)	201 (47.3%)	112 (46.9%)	
Sex				0.147
Female	284 (40.3%)	185 (43.5%)	113 (47.3%)	
Male	421 (59.7%)	240 (56.5%)	126 (52.7%)	
Smoking history				0.730
No	520 (73.8%)	322 (75.8%)	180 (75.3%)	
Yes	185 (26.2%)	103 (24.2%)	59 (24.7%)	
Drinking history				0.111
No	598 (84.8%)	340 (80.0%)	199 (83.3%)	
Yes	107 (15.2%)	85 (20.0%)	40 (16.7%)	
CEA (ng/mL)				0.596
<5	505 (71.6%)	299 (70.4%)	163 (68.2%)	
≥5	200 (28.4%)	126 (29.6%)	76 (31.8%)	
T stage				0.071
T1-2	93 (13.2%)	76 (17.9%)	31 (13.0%)	
T3-4	612 (86.8%)	349 (82.1%)	208 (87.0%)	
N stage				0.428
0	369 (52.3%)	215 (50.6%)	122 (51.0%)	
1	186 (26.4%)	133 (31.3%)	70 (29.3%)	
2	150 (21.3%)	77 (18.1%)	47 (19.7%)	
pTNM				0.153
1	74 (10.5%)	62 (14.6%)	25 (10.5%)	
2	295 (41.8%)	153 (36.0%)	97 (40.6%)	
3	336 (47.7%)	210 (49.4%)	117 (49.0%)	
Pathological type				0.197
Adenocarcinoma	616 (87.4%)	373 (87.8%)	219 (91.6%)	
Other	89 (12.6%)	52 (12.2%)	20 (8.4%)	
Differentiation grade				0.895
Poorly differentiated	81 (11.5%)	45 (10.6%)	27 (11.3%)	
Moderately/Well differentiated	624 (88.5%)	380 (89.4%)	212 (88.7%)	
Perineural invasion (PNI)				0.307
Absent	568 (80.6%)	330 (77.6%)	183 (76.6%)	
Present	137 (19.4%)	95 (22.4%)	56 (23.4%)	
Lymphovascular invasion (LVI)				0.225
Absent	488 (69.2%)	303 (71.3%)	155 (64.9%)	
Present	217 (30.8%)	122 (28.7%)	84 (35.1%)	
Postoperative adjuvant therapy				0.055
No	185 (26.2%)	140 (32.9%)	69 (28.9%)	
Yes	520 (73.8%)	285 (67.1%)	170 (71.1%)	

Data are n (%) unless otherwise specified.

* Data are medians, with interquartile ranges in square brackets.

** P values refer to comparisons among the three cohorts.

CEA, carcinoembryonic antigen. Postoperative adjuvant therapy indicates documented receipt of any postoperative systemic therapy; treatment-initiation dates, regimens, dose intensity, cycle number and duration were not harmonised across centres.

Reverse Kaplan–Meier median follow-up was 31, 34 and 36 months in the training cohort and external test cohorts 1 and 2, respectively. Numbers at risk and recurrence patterns at 12, 24 and 36 months are provided in Supplementary Table S4 and Supplementary Figure S2. Potential early loss to follow-up occurred in 28.4%, 27.8% and 15.9% of patients, respectively. After inverse probability of censoring weighting, the imaging scores remained independently associated with recurrence-free survival, and risk-group separation persisted in all cohorts (Supplementary Figure S3; Supplementary Table S5). The first recurrence was local in 63 patients, distant in 238 and synchronous local and distant in 31. Both Tumor DL and VAT DL scores were associated with local and distant recurrence, without significant heterogeneity between recurrence types. Risk groups also stratified the cumulative incidence of both outcomes, with similar findings after excluding synchronous events (Supplementary Table S6; Supplementary Figure S4).

### Development and performance of single-modality deep learning models

Two single-modality models were developed to generate continuous recurrence-risk scores: a tumour DL model using tumour-centred CT volumes and a VAT DL model using VAT-containing CT slices. Using cut-offs derived from the training cohort, both models stratified RFS across all datasets (all log-rank P < 0.001; Supplementary Figure S5). The discrimination performance of the single-modality models is shown in Table 2 and Figure 3. Both models showed prognostic discrimination in the independent external test cohorts. The tumour DL model achieved Harrell’s C-indices of 0.681 (95% CI, 0.624–0.739) and 0.717 (95% CI, 0.647–0.784) in external test cohorts 1 and 2, respectively, with corresponding 2-year time-dependent AUCs of 0.665 (95% CI, 0.592–0.736) and 0.748 (95% CI, 0.656–0.828). The VAT DL model yielded C-indices of 0.671 (95% CI, 0.613–0.732) and 0.708 (95% CI, 0.635–0.780), with corresponding 2-year time-dependent AUCs of 0.648 (95% CI, 0.577–0.726) and 0.725 (95% CI, 0.634–0.814), respectively.

**TABLE 2:** Comparison of performance among different models.

Model	C-index (95% CI)	2-year time-dependent AUC (95% CI)
Training cohort		
Clinical-only	0.707 (0.662–0.746)	0.701 (0.647–0.751)
Tumor DL	0.775 (0.740–0.809)	0.803 (0.757–0.848)
VAT DL	0.777 (0.733–0.816)	0.800 (0.750–0.846)
Tumor + VAT	0.827 (0.796–0.856)	0.852 (0.809–0.893)
Postoperative multimodal	0.843 (0.813–0.871)	0.870 (0.829–0.908)
External test cohort 1		
Clinical-only	0.709 (0.655–0.761)	0.746 (0.680–0.808)
Tumor DL	0.681 (0.624–0.739)	0.665 (0.592–0.736)
VAT DL	0.671 (0.613–0.732)	0.648 (0.577–0.726)
Tumor + VAT	0.734 (0.679–0.787)	0.711 (0.638–0.780)
Postoperative multimodal	0.772 (0.718–0.822)	0.757 (0.687–0.819)
External test cohort 2		
Clinical-only	0.715 (0.652–0.777)	0.737 (0.650–0.812)
Tumor DL	0.717 (0.647–0.784)	0.748 (0.656–0.828)
VAT DL	0.708 (0.635–0.780)	0.725 (0.634–0.814)
Tumor + VAT	0.772 (0.712–0.827)	0.807 (0.734–0.875)
Postoperative multimodal	0.810 (0.760–0.859)	0.836 (0.766–0.899)

AUC, area under the time-dependent receiver operating characteristic curve; CI, confidence interval; DL, deep learning; VAT, visceral adipose tissue. Two-year AUCs, were estimated using inverse probability of censoring weighting.

**FIGURE 3:**
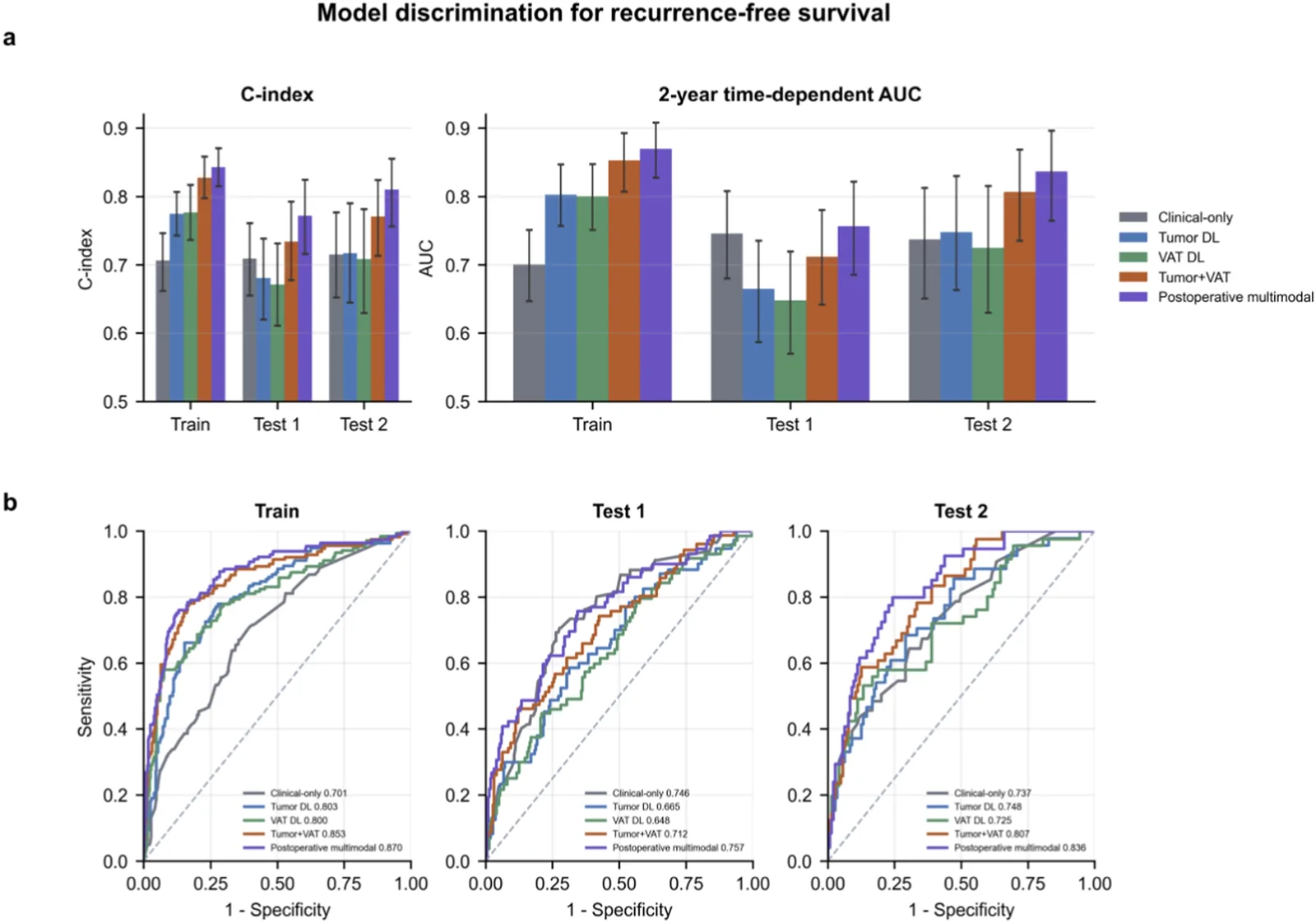
Performance of the clinicopathological, single-modality, and multimodal recurrence-risk models. **(a)** Bar plots compare Harrell’s C-index and the 2-year cumulative/dynamic time-dependent AUC across the training cohort and two external test cohorts. Error bars indicate bootstrap 95% confidence intervals. Models included the clinicopathological-only model, tumour DL model, VAT DL model, preoperative Tumor + VAT model, and postoperative multimodal model. **(b)** Two-year time-dependent receiver operating characteristic curves were estimated using inverse probability of censoring weighting.

### Reproducibility and sensitivity analyses

The workflow audit confirmed consistent 32-slice VAT sampling across all cohorts, with no slice duplication among the 1,369 patients. Alternative CT windows preserved score ranking (Spearman’s rho ≥ 0.972) and final-model discrimination. C-indices ranged from 0.839 to 0.844 in the training cohort, 0.767–0.772 in external test cohort 1 and 0.804–0.810 in external test cohort 2 (Supplementary Table S7; Supplementary Figure S6). In the 30-case observer study, the median interobserver and intraobserver Dice coefficients were both 0.984. The Tumor DL score showed good reproducibility, with corresponding intraclass correlation coefficients (ICCs) of 0.862 and 0.889. Tumour-segmentation variation had limited influence on the final-model linear predictor (ICC, 0.953; 95% confidence interval [CI], 0.878–0.985). Interobserver and intraobserver agreement for the three risk categories was 76.7%, with weighted kappa values of 0.734 and 0.761, respectively (Supplementary Figure S7). However, fixed-physical-field tumour preprocessing and positional encoding of VAT slices yielded lower discrimination than the primary models (Supplementary Table S8; Supplementary Figure S8).

Repeated five-fold cross-validation showed appreciable variability, with mean fold-level C-indices of 0.556 for Tumor DL and 0.498 for VAT DL. Complete-risk-set Cox-head refitting did not materially alter external discrimination, and the 95% CIs for all paired differences included zero (Supplementary Table S9; Supplementary Figure S9). Compared with the clinicopathological-only model, the postoperative multimodal model improved the C-index by 0.136 (95% CI, 0.092–0.178), 0.062 (95% CI, −0.010–0.132) and 0.095 (95% CI, 0.023–0.168) in the training cohort and external test cohorts 1 and 2, respectively. Improvement was significant in the training cohort and external test cohort 2 but not consistently significant in external test cohort 1 (Supplementary Table S10; Supplementary Figure S10).

### Time-dependent discrimination and prediction error

At 1, 2 and 3 years, the postoperative multimodal model achieved time-dependent area under the curve (AUC) values of 0.870, 0.870 and 0.875 in the training cohort; 0.808, 0.757 and 0.827 in external test cohort 1; and 0.849, 0.836 and 0.840 in external test cohort 2, respectively. Corresponding Brier scores were 0.069, 0.103 and 0.112; 0.066, 0.129 and 0.156; and 0.085, 0.130 and 0.176, respectively. The integrated Brier scores over 1–36 months were 0.080, 0.092 and 0.099 across the three cohorts (Supplementary Table S11; Supplementary Figure S11).

### Cox regression and postoperative multimodal model

Univariable and multivariable Cox regression results are presented in Table 3. After adjustment for clinicopathological variables, both Tumor DL (hazard ratio [HR], 2.016; 95% confidence interval [CI], 1.694–2.399) and VAT DL scores (HR, 2.099; 95% CI, 1.808–2.436) remained independent predictors of recurrence-free survival (both P < 0.001). N stage and perineural invasion also retained independent prognostic value.

**TABLE 3:** Univariate and multivariate Cox regression analysis for predicting postoperative recurrence in the training cohort.

Characteristics	Univariate analysis		Multivariate analysis	
	HR (95% CI)	P value	HR (95% CI)	P value
Age	1.130 (0.970–1.317)	0.118		
Sex	0.948 (0.700–1.282)	0.727		
Smoking history	1.100 (0.789–1.535)	0.573		
Drinking history	0.960 (0.628–1.467)	0.850		
Tumour location	1.078 (0.800–1.453)	0.622		
CEA	1.538 (1.124–2.104)	0.007	1.246 (0.905–1.714)	0.177
T stage	2.320 (1.291–4.172)	0.005	1.018 (0.553–1.872)	0.955
N stage	2.006 (1.674–2.403)	<0.001	1.488 (1.217–1.820)	<0.001
Pathological type	1.943 (1.340–2.815)	<0.001	0.771 (0.513–1.158)	0.211
Differentiation grade	0.585 (0.398–0.860)	0.006	1.270 (0.823–1.960)	0.280
Perineural invasion (PNI)	2.084 (1.508–2.880)	<0.001	1.507 (1.015–2.238)	0.042
Lymphovascular invasion (LVI)	2.549 (1.891–3.436)	<0.001	1.002 (0.700–1.433)	0.993
VAT DL score	2.522 (2.221–2.864)	<0.001	2.099 (1.808–2.436)	<0.001
Tumor DL score	2.645 (2.261–3.094)	<0.001	2.016 (1.694–2.399)	<0.001

The postoperative multimodal model integrated both imaging scores with seven clinicopathological variables. It achieved C-indices of 0.772 and 0.810 and 2-year time-dependent area under the curve values of 0.757 and 0.836 in external test cohorts 1 and 2, respectively. Calibration was generally acceptable, although short-horizon underprediction was observed in both external cohorts without recalibration (Supplementary Table S12). Survival decision-curve analysis demonstrated positive net benefit across the prespecified 5%–30% threshold range, although the incremental benefit over clinicopathological and pathological tumour-node-metastasis stage models varied by horizon and threshold (Figure 4; Supplementary Table S13; Supplementary Figure S12). Ridge-penalised modelling produced performance similar to the original model, with low multicollinearity and no evidence of proportional-hazards violations (Supplementary Table S14; Supplementary Figure S13). The final risk score remained independently prognostic after adjustment for adjuvant therapy, stage and cohort, with no significant score-by-treatment interaction (Supplementary Table S15; Supplementary Figure S14). Detailed results are provided in the Supplementary Results. Additional ablation and training-trajectory analyses are provided in Supplementary Table S16 and Supplementary Figures S15 and S16.

**FIGURE 4:**
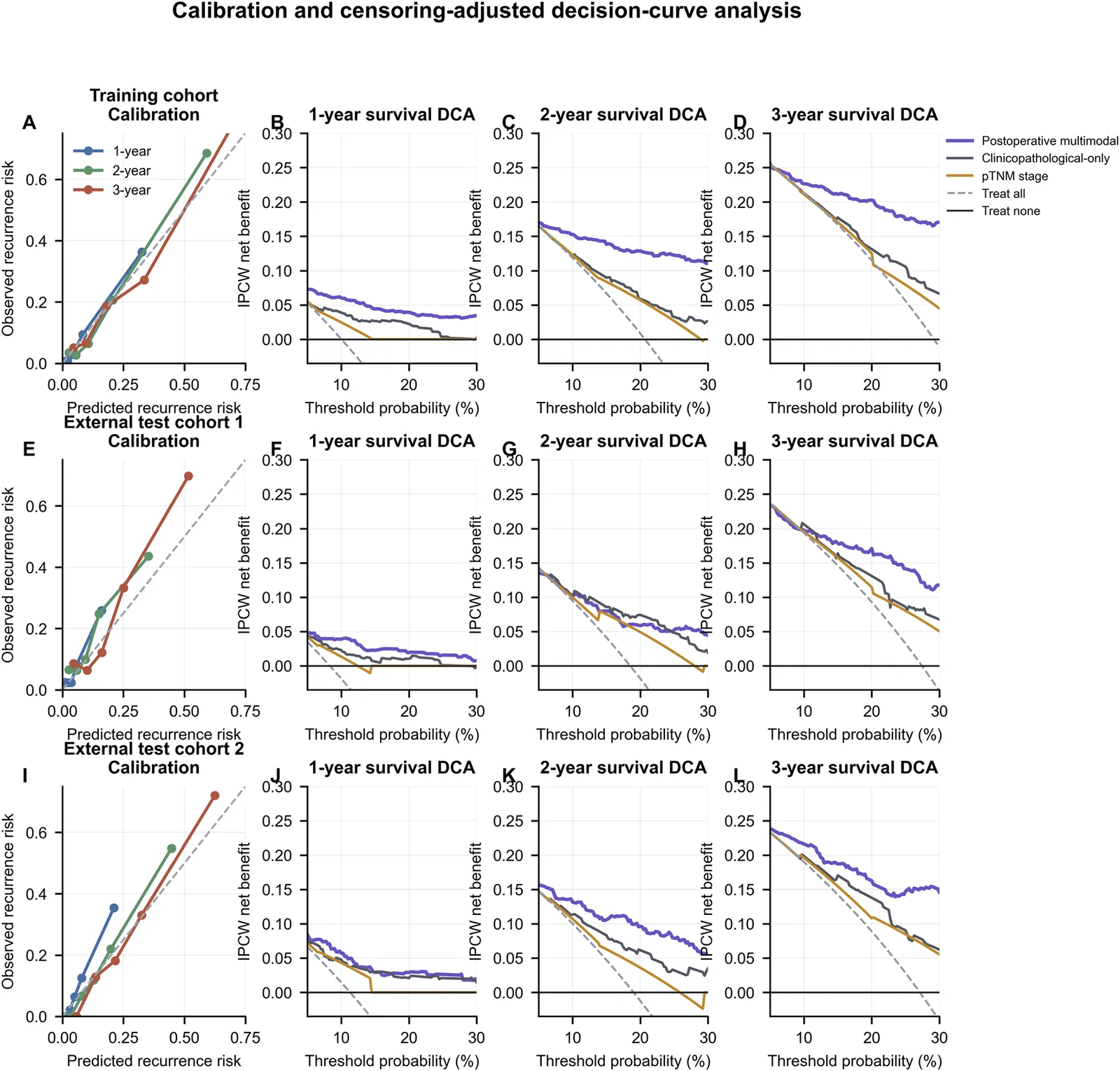
Calibration and censoring-adjusted survival decision-curve analysis. **(A, E, I)** Calibration of the postoperative multimodal model at 1, 2, and 3 years in the training cohort and two external test cohorts. Observed recurrence risks were estimated by Kaplan-Meier analysis. **(B–D, F–H, J–L)** IPCW-adjusted survival decision curves at 1, 2, and 3 years comparing the postoperative multimodal model, clinicopathological-only model, pTNM stage model, treat-all strategy, and treat-none strategy across the prespecified 5%–30% threshold-probability range. Model coefficients and Breslow baseline survival functions were estimated in the training cohort and locked for external evaluation. DCA, decision-curve analysis; IPCW, inverse probability of censoring weighting.

### Risk stratification and model interpretation

Using the training-derived final-risk-score cut-offs of 0.485813 and 1.560461, patients were classified as low, intermediate or high risk; the cut-offs were applied unchanged to both external cohorts (Figure 5). Relative to the low-risk group, intermediate-risk hazard ratios were 2.77 (95% CI, 1.45–5.29), 3.11 (95% CI, 1.45–6.69) and 3.72 (95% CI, 1.21–11.50), and high-risk hazard ratios were 16.01 (95% CI, 9.03–28.39), 12.55 (95% CI, 5.96–26.40) and 18.14 (95% CI, 6.14–53.65) in the training cohort and external test cohorts 1 and 2, respectively. Because no recurrence occurred in the low-risk group of external test cohort 2, its hazard ratios were estimated using ridge-penalised Cox regression and should be interpreted cautiously. Overall log-rank tests were significant in all cohorts, and all pairwise comparisons remained significant after Holm correction (all adjusted P < 0.003; Supplementary Table S12).

**FIGURE 5:**
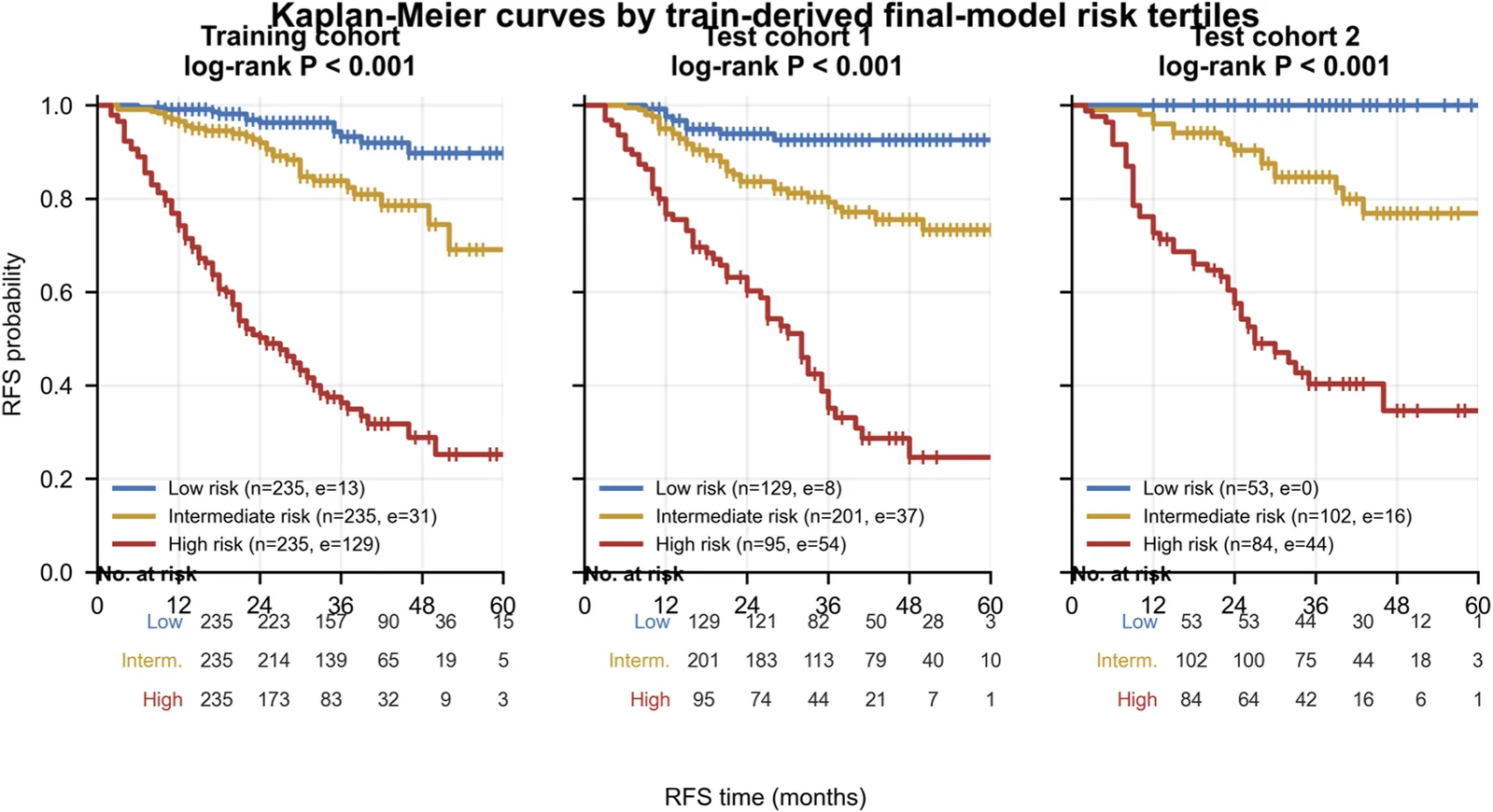
Kaplan-Meier recurrence-free survival curves for the postoperative multimodal recurrence-risk model. Patients were stratified into low-, intermediate- and high-risk groups using training-derived final-risk-score cut-offs of 0.485813 and 1.560461, which were applied unchanged to both external cohorts. Curves are shown for the training cohort and two external test cohorts. Vertical tick marks indicate censored observations, and numbers at risk are provided at 0, 12, 24, 36, 48 and 60 months. Overall log-rank tests were significant in all cohorts, and all pairwise comparisons remained significant after Holm correction (all adjusted P < 0.003; Supplementary Table S12).

Representative cases from these risk strata are shown in Figure 6. Patients with rapid recurrence generally had higher final multimodal risk scores, whereas non-recurrent cases generally had lower scores. Tumour Grad-CAM and VAT attention maps qualitatively indicated image regions contributing to individual model outputs.

**FIGURE 6:**
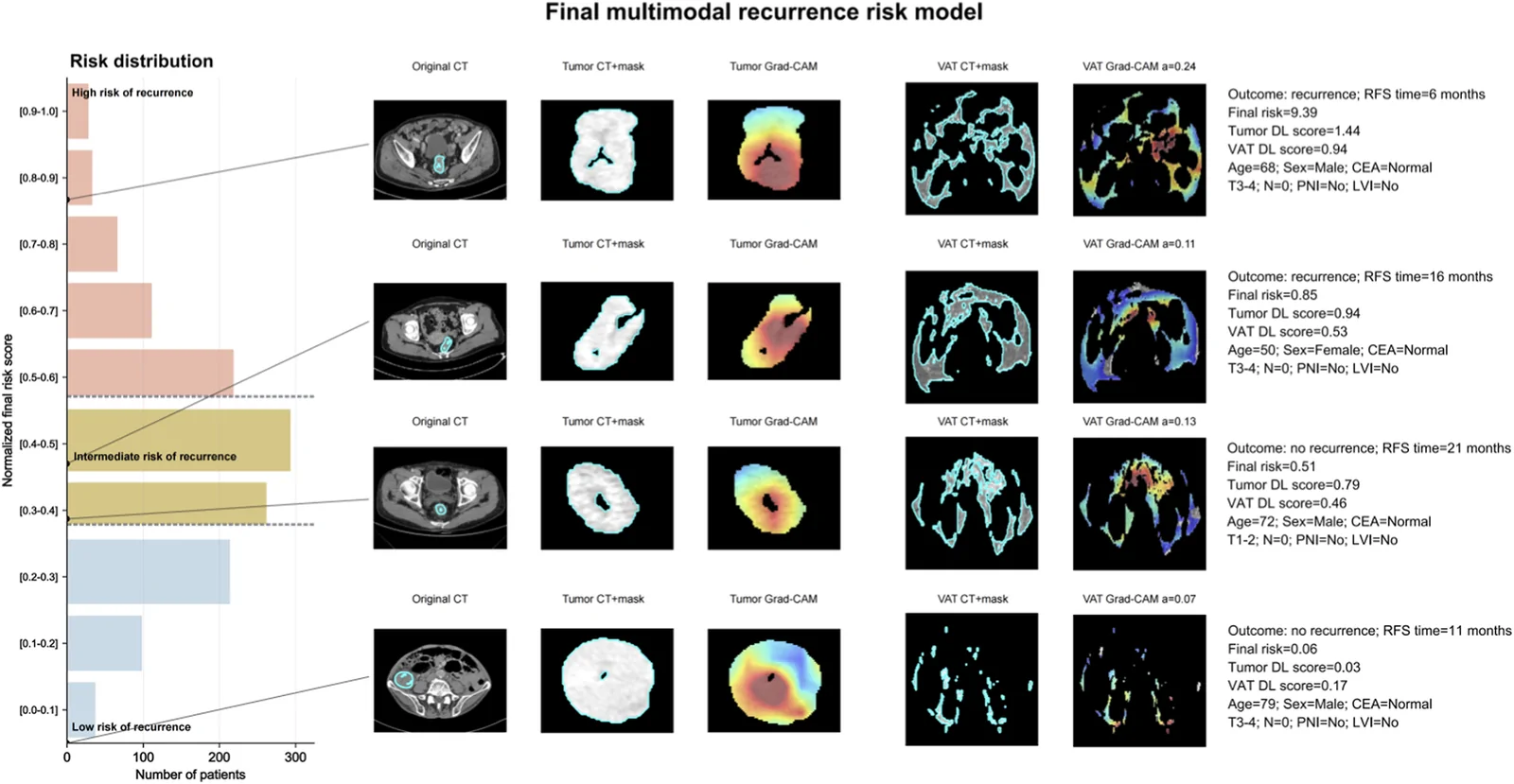
Purposefully selected illustrative cases of multimodal recurrence-risk prediction. The left panel shows the distribution of normalised final risk scores and the training-derived low-, intermediate- and high-risk strata. Four cases were selected after model evaluation to span the risk distribution, include recurrent and non-recurrent outcomes, and represent different cohorts where possible; complete CT images, tumour and VAT masks and corresponding maps were required. Original CT images, tumour CT masks, tumour Grad-CAM maps, VAT CT masks and VAT attention maps are shown together with patient-level clinical information and model scores. The cases were not randomly sampled and were used only for qualitative illustration. The maps indicate regions contributing to individual model outputs and do not constitute biological or population-level validation.

## Discussion

Accurate postoperative recurrence-risk assessment is important for tailoring surveillance and multidisciplinary treatment planning in colorectal cancer (CRC). In this multicentre study, the preoperative Tumor + VAT CT model provided non-invasive risk stratification, while incorporating postoperative clinicopathological variables further improved discrimination. The postoperative multimodal model achieved C-indices of 0.772 and 0.810 and 2-year time-dependent areas under the curve of 0.757 and 0.836 in two independent external cohorts. Both imaging scores remained independently associated with recurrence-free survival after adjustment for pathological factors. Time-dependent discrimination and prediction-error analyses confirmed model performance during the first 3 postoperative years. Together, these findings demonstrate that tumour-centred and host-related CT phenotypes provide complementary prognostic information and support the clinical potential of multimodal recurrence-risk assessment.

The prognostic value of the Tumor DL score is consistent with previous CT radiomics and deep-learning studies showing that quantitative imaging captures tumour phenotypes beyond conventional staging (Badic et al., 2022; Liu et al., 2021). Unlike handcrafted radiomics, which depends on predefined descriptors, a three-dimensional convolutional neural network learns hierarchical representations directly from volumetric images (Zhou et al., 2025b). The tumour branch therefore extracted prognostically informative patterns reflecting attenuation heterogeneity, invasive margins and stromal characteristics that are incompletely represented by pathological stage. MRI radiomics studies have similarly demonstrated that quantitative features complement visual staging in characterising local rectal tumour aggressiveness (Patane et al., 2025). Although the histological and molecular basis of these learned features remains to be established, the independent contribution of the Tumor DL score confirms that tumour-centred CT contains prognostic information beyond conventional clinicopathological variables.

The VAT DL score showed prognostic strength comparable to the Tumor DL score, highlighting the contribution of host phenotype to recurrence risk. The seed-and-soil hypothesis and subsequent work on tumour-stroma interactions emphasise that metastasis depends on both malignant cells and a permissive host environment (Paget, 1889; Langley and Fidler, 2011). Visceral adipose tissue is a metabolically active endocrine and immune organ that releases adipokines, inflammatory cytokines and metabolic mediators linked to insulin resistance, angiogenesis and immune dysregulation (Khandekar et al., 2011; Chaplin et al., 2022; Riondino et al., 2014). CT-derived visceral adiposity has also been associated with metastasis and survival in CRC (Park et al., 2022). Our approach advances conventional single-slice measurements by modelling VAT throughout the scanned abdomen. Attention-based multiple-instance learning aggregates slice-level information using patient-level outcome labels (Ilse et al., 2018; Yao et al., 2020), enabling the model to identify prognostically informative VAT patterns without predefined anatomical selection. These findings establish whole-abdominal VAT as a complementary imaging phenotype for recurrence-risk assessment.

Competing-risk analyses showed that both imaging scores were associated with local recurrence and distant metastasis, without significant heterogeneity between recurrence types. This demonstrates broad prognostic relevance across major recurrence patterns, although the limited number of local events in external test cohort 1 reduced statistical precision. The postoperative model further combined imaging scores with pathological variables, among which N stage and perineural invasion retained independent prognostic value. These variables represent lymphatic dissemination and neural invasion, both established adverse features in CRC (Knijn et al., 2016; Yang et al., 2015). The Tumor + VAT model therefore provides preoperative imaging-based risk assessment, whereas the Tumor + VAT + Clinical model supports postoperative stratification after pathological review. Compared with the clinicopathological-only model, the multimodal model provided clear incremental performance in the training cohort and external test cohort 2, with more modest improvement in external test cohort 1.

Censoring-adjusted decision-curve analysis demonstrated positive net benefit across prespecified thresholds of 5%–30%, supporting the potential value of the postoperative multimodal model for risk-informed clinical assessment. The magnitude of improvement over clinicopathological and pathological tumour-node-metastasis stage models varied by horizon and threshold, and short-horizon underprediction was observed in both external cohorts without recalibration. The training-derived tertiles nevertheless achieved consistent prognostic stratification across cohorts, although they were designed as exploratory risk categories rather than intervention thresholds. Treatment-adjusted and overlap-weighted analyses confirmed that the final score remained prognostic in patients with and without documented adjuvant therapy. These analyses support the robustness of the prognostic association but do not demonstrate treatment-benefit prediction. The all-candidate ridge model produced highly concordant scores and similar discrimination, demonstrating that model performance was not driven solely by univariable variable selection. Low variance inflation factors and nonsignificant Schoenfeld-residual tests further supported the stability of the fitted Cox model.

Gradient-weighted class activation mapping (Grad-CAM) and VAT attention maps provided qualitative localisation of regions contributing to model predictions (Selvaraju et al., 2017). Tumour maps highlighted intratumoural and peritumoural regions, whereas VAT maps demonstrated spatially heterogeneous activation across abdominal adipose tissue. External validation across two centres with heterogeneous voxel-spacing distributions further supports model transportability. Discrimination was retained in both colon and rectal cancer, with no significant interaction by tumour location, centre, sex or adjuvant therapy. The stage interaction indicates that the magnitude of the prognostic association varies across disease stages. Because patients receiving preoperative treatment were excluded, the present findings apply specifically to patients undergoing upfront curative-intent surgery and should not be extrapolated directly to locally advanced rectal cancer treated with neoadjuvant approaches.

Several limitations warrant emphasis. The retrospective design remains susceptible to selection and follow-up bias; with median follow-up of 31–36 months and some early censoring, late recurrence may have been incompletely captured. Follow-up performed elsewhere could not be distinguished consistently from true loss to follow-up, and although inverse probability of censoring weighting supported the primary findings, residual bias cannot be excluded. Harmonised death dates and organ-specific metastatic sites were unavailable, limiting competing-risk analyses to local versus distant first recurrence. Manual tumour segmentation also limits scalability; despite good reproducibility in 30 cases, annotation subjectivity remains, supporting future comparison with automated segmentation. Model performance depended partly on index-space tumour cropping and VAT sampling without positional encoding, while repeated five-fold training showed lower and more variable internal performance than the original split. Although alternative CT windows and complete-risk-set Cox-head refitting largely preserved external risk ranking, prospective validation using harmonised preprocessing, nested resampling and risk-set-consistent training is required. Finally, reliance on a single preoperative CT prevented assessment of longitudinal changes. Radiogenomic and radiopathomic analyses, including circulating tumour DNA integration (Tie et al., 2022), delta-radiomics (Nardone et al., 2025) and liquid-biopsy integration (Belfiore et al., 2025) may clarify the biological and longitudinal relevance of these imaging signatures. Overall, tumour and whole-abdominal VAT phenotypes provided complementary prognostic information, and postoperative pathological variables further strengthened recurrence-risk prediction. This framework provides a strong basis for prospective validation across contemporary treatment pathways and clinically actionable risk thresholds.

## Data Availability

The original contributions presented in the study are included in the article/Supplementary Material, further inquiries can be directed to the corresponding author.
