# Supplementary material for: Preoperative CT deep learning of tumor and visceral adipose tissue combined with postoperative pathology predicts colorectal cancer recurrence: a multicenter study
Source: Front Mol Biosci. 2026 Sep 24;13:1937172. doi: 10.3389/fmolb.2026.1937172 (PMC13646159; doi:10.3389/fmolb.2026.1937172)
Supplement: Supplementary file 1 [file DataSheet1.docx]

**Supplementary Material**

# Supplementary Methods

## Follow-up and recurrence assessment

Local recurrence was defined as recurrence at the surgical incision, anastomotic site, pelvis, or original operative field. Distant metastasis included recurrence in the peritoneum, liver, lung, lymph nodes, brain, bone, or other organs. For patients without documented recurrence, censoring occurred at the last assessment at which recurrence status could be established by imaging, endoscopy, pathological examination, or documented clinical follow-up. Patients who developed recurrence before 24 months were classified as events and were not considered potentially lost to follow-up.

Follow-up duration was estimated using the reverse Kaplan–Meier method, treating recurrence events as censored observations and recurrence-free censoring as follow-up events. Follow-up adequacy was described using the median and interquartile range, observed minimum–maximum duration, numbers at risk at 12, 24, and 36 months, and cumulative first recurrences classified as local only, distant only, or synchronous local and distant recurrence. Synchronous events were classified as distant metastasis in the primary competing-risk analysis and excluded in a sensitivity analysis.

Confirmed loss to follow-up could not be distinguished consistently from follow-up conducted outside the participating centres. Potential early loss to follow-up was therefore conservatively defined as recurrence-free censoring before 24 months. Harmonised death status and date fields were unavailable; consequently, death before recurrence could not be distinguished reliably from other recurrence-free censoring and was not modelled as a separate competing event. The competing-risk analysis was restricted to local versus distant first recurrence.

## Tumour and VAT segmentation

Primary tumour regions were manually delineated slice by slice on portal venous phase images by Reader A, an abdominal radiologist with 12 years of experience. A senior abdominal radiologist with 35 years of experience reviewed all tumour masks, and discrepancies were resolved by consensus. VAT was automatically segmented on a graphics processing unit using TotalSegmentator version 2.13.0, the tissue_4_types task and default inference configuration; the torso_fat output was retained as the VAT mask (18). Reader A performed case-level visual quality control. No voxel-level manual correction was applied to any VAT mask used for model development or evaluation (0/1,369 patients). All segmentation and quality-control procedures were performed without access to recurrence outcomes, follow-up data or model predictions.

For the reproducibility analysis, 30 patients were randomly selected. A second abdominal radiologist with 12 years of experience independently delineated the primary tumour for interobserver assessment. Reader A repeated the delineation after one month for intraobserver assessment without access to the original masks. Mask-level agreement was quantified using the Dice similarity coefficient, Jaccard index and 95th-percentile Hausdorff distance (HD95). The trained tumour 3D CNN was then applied to the original, interobserver and intraobserver segmentations without retraining. Tumor DL-score stability was evaluated using agreement analyses and intraclass correlation coefficients, as reported in Figure S7.

## Image preprocessing and deep learning model development

### Image preprocessing

CT attenuation values were clipped to an abdominal soft-tissue window of −200 to 300 HU and linearly normalised to 0–1 before model input. No scanner-specific harmonisation or ComBat correction was applied, allowing the two external cohorts to remain unchanged for the assessment of model transportability.

For the tumour branch, images were processed in native array space without resampling to a common physical voxel spacing. A three-dimensional bounding box enclosing the tumour mask was expanded by 12 voxels along each axis. This represented an index-based margin rather than a fixed physical-distance margin. The cropped volume was resized to 80 × 80 × 80 voxels using trilinear interpolation for CT images and nearest-neighbour interpolation for binary masks. The input comprised two channels: the masked CT volume and the binary tumour mask.

For the VAT branch, axial slices containing more than 50 VAT-mask pixels were considered valid. Thirty-two slices were selected deterministically at evenly spaced quantiles between the 5th and 95th percentiles of valid craniocaudal positions. Selected slices were stored in anatomical order, and the same sampling procedure was applied to the training, internal-validation and external-test cohorts. Each slice was cropped around the VAT mask using a 16-pixel in-plane margin and resized to 160 × 160 pixels with bilinear interpolation for CT and nearest-neighbour interpolation for the mask. The two input channels were the masked CT slice and binary VAT mask.

A terminal-slice padding procedure was predefined for patients with fewer than 32 valid VAT slices. However, all 1,369 patients had at least 57 valid slices; therefore, no padding or slice duplication was required. The primary attention-MIL model did not receive explicit anatomical coordinates. Although slice order was retained in the stored tensor, attention pooling was mathematically permutation invariant. Because both branches used pre-generated cached tensors, no online geometric or intensity augmentation was applied after cache loading.

### Preprocessing sensitivity analyses

Original CT voxel spacing and physical tumour bounding-box dimensions were audited in all 1,369 patients. To evaluate the effect of physical-space standardisation, the Tumor 3D CNN was retrained using a 2.5-mm isotropic grid and a fixed 96 × 96 × 96 matrix, corresponding to a 240-mm cubic field of view centred on the tumour. This field of view was determined using only the development cohort. The largest tumour dimension in that cohort was 175.5 mm, leaving more than the prespecified 12-mm boundary on each side. CT images and masks were resampled using linear and nearest-neighbour interpolation, respectively. The network architecture, loss function, optimiser and random seed were otherwise unchanged, and checkpoint selection used only the internal-validation subset.

A position-aware VAT sensitivity model was also developed. Each slice’s normalised craniocaudal coordinate within the sampled 5th–95th-percentile range was embedded and added to its 64-dimensional feature representation before attention pooling. All other inputs and training procedures were unchanged. Primary and sensitivity models were compared using paired patient-level bootstrap confidence intervals.

### Network architecture

The tumour branch consisted of a residual 3D CNN. A 7 × 7 × 7 convolutional stem was followed by four residual blocks with output widths of 10, 20, 40 and 40 channels. Each residual block contained two 3 × 3 × 3 convolutions, with downsampling performed in the second and third blocks. Adaptive global average pooling was followed by a 48-dimensional fully connected layer, dropout with a rate of 0.55 and a linear risk head that generated the Tumor DL score.

The VAT branch used a shared 2D CNN to encode individual VAT slices and attention-based multiple-instance learning to generate a patient-level representation (16). The encoder comprised four convolutional blocks with 16, 32, 64 and 64 channels, followed by adaptive global average pooling and a 64-dimensional slice embedding h_i. Attention logits were calculated as

uᵢ = wᵀ tanh(Vhᵢ + b)

aᵢ = exp(uᵢ) / Σⱼ exp(uⱼ)

z = Σᵢ aᵢhᵢ

### Cox loss implementation

Both branches were trained using time-to-event supervision. For mini-batch B, predicted log-risk r_i, event indicator \delta_i and observed time t_i, the implemented loss was

Lᴮ = −[Σᵢ∈ᴮ δᵢ]⁻¹ Σᵢ∈ᴮ:δᵢ=1 {rᵢ − log[Σⱼ∈ᴮ:tⱼ≥tᵢ exp(rⱼ)]}.

Patients were ordered by decreasing observed time within each randomly shuffled mini-batch, and the denominator was calculated using the log-cumulative-sum-exp operation. Risk sets were therefore restricted to patients within the same mini-batch, representing a stochastic approximation to the full Cox partial likelihood.

### Model training and checkpoint selection

The 705-patient development cohort, which included 173 recurrence events, was divided into an internal training subset of 564 patients (138 events) and an internal-validation subset of 141 patients (35 events). The 80:20 split was stratified by recurrence status using random seed 2026. Both branches were trained using AdamW and CUDA automatic mixed precision with constant learning rates.

For the Tumor DL model, the batch size was 6, learning rate was 8 × 10⁻⁵, weight decay was 2 × 10⁻⁴, dropout was 0.55 and the maximum training duration was 25 epochs. Early stopping was based on internal-validation C-index with a patience of six epochs. Training stopped after 13 epochs, and the checkpoint from epoch 5 was retained.

For the VAT DL model, the batch size was 8, learning rate was 1 × 10⁻⁴, weight decay was 5 × 10⁻⁵, dropout was 0.35 and the maximum training duration was 35 epochs. No early stopping was applied, and the checkpoint from epoch 29 was retained. Candidate checkpoints were ranked according to internal-validation C-index and, as prespecified in the original development procedure, were retained only when the apparent C-index in the complete development cohort was between 0.70 and 0.80.

The external cohorts were not used for data splitting, checkpoint selection, feature standardisation, hyperparameter tuning or risk cut-off determination.

### Internal stability and complete-risk-set sensitivity analyses

Internal model stability was assessed using three repeats of five-fold cross-validation within the development centre. Splits were jointly stratified by recurrence status and follow-up-time quartile within each status group. Repeat-level split seeds were 2026, 2126 and 2226, with separate fold-specific training seeds. Each model was reinitialised and trained from scratch for the fixed primary checkpoint duration of five epochs for the Tumor DL model and 29 epochs for the VAT DL model. Held-out folds were not used for checkpoint selection, and both external cohorts remained inaccessible. Fold-level and pooled out-of-fold C-indices and 2-year cumulative/dynamic AUCs were calculated.

To assess the influence of the mini-batch risk-set approximation, locked 48-dimensional Tumor and 64-dimensional VAT feature representations were extracted. Constant feature channels were identified using only the internal training subset and removed. The remaining features were standardised, and a ridge Cox head was selected using the fixed internal-validation subset. The selected head was subsequently refitted in all 705 development patients using complete development-cohort risk sets and Breslow handling of tied event times. Model parameters were then locked before evaluation in the external cohorts.

## Locked external inference pipeline

For external inference, all preprocessing parameters, model weights, score-standardisation statistics, Cox coefficients, the Breslow baseline hazard and risk-group cut-offs were locked from the development cohort. Tumour volumes were windowed, cropped with the 12-voxel index-space margin, resized to 80 x 80 x 80 and passed to the locked 3D CNN. VAT masks were used to select 32 ordered quantile slices; each slice was cropped, resized to 160 x 160 and encoded by the shared 2D CNN before softmax attention pooling. No test-time augmentation, checkpoint ensembling or external-centre parameter updating was used.

Tumor and VAT scores were standardised using development-cohort means and standard deviations and entered into the locked preoperative imaging Cox model. The postoperative model additionally used CEA, ordinal N stage, pathological type, differentiation grade, PNI, LVI and dichotomised T stage. Absolute recurrence risk was calculated as 1 - S0(t)^exp(eta), where S0(t) is the development-cohort Breslow baseline survival and eta is the locked linear predictor. Training-derived cut-offs of 0.485813 and 1.560461 assigned low-, intermediate- and high-risk groups.

## Model evaluation and interpretation

Discrimination was assessed using Harrell’s C-index and cumulative/dynamic time-dependent area under the curve (AUC) at 12, 24 and 36 months. Time-dependent AUCs were estimated using inverse probability of censoring weighting (IPCW), with the censoring distribution estimated from the training cohort. Prediction error was evaluated using IPCW Brier scores at the same time points and the integrated Brier score (IBS) over 1–36 months.

Individual survival probabilities were calculated from the training-cohort Breslow baseline hazard and each locked model linear predictor. No model was retrained or recalibrated in either external cohort. Non-parametric patient-level bootstrap resampling with 1,000 iterations was used to derive 95% confidence intervals for time-dependent AUC, Brier score and IBS estimates. The prespecified 2-year comparison is presented in Figure 3; complete results at 12, 24 and 36 months and prediction-error curves are provided in Table S11 and Figure S11.

Gradient-weighted class activation mapping (Grad-CAM) was used for qualitative interpretation of the imaging models (20). For the tumour branch, Grad-CAM heatmaps were overlaid on tumour-centred CT slices to identify intratumoural and peritumoural regions contributing to the Tumor DL score. For the VAT branch, attention weights identified the most informative anatomical slices, and the corresponding heatmaps highlighted adipose regions contributing to the VAT DL score. These maps indicate regions influencing model output but do not establish biological mechanisms or causal associations with recurrence.

The four cases shown in Figure 6 were purposefully selected after completion of model evaluation to span the final-model risk distribution, include recurrent and non-recurrent outcomes, and represent the development and external cohorts where possible. Eligible cases were required to have complete CT images, tumour and VAT masks and corresponding visualisation maps. Cases with severe artefacts that prevented visual interpretation were excluded. Representative cases were used solely for qualitative illustration and did not contribute to model development, checkpoint selection, cut-off determination or performance evaluation.

## Statistical analysis

Continuous variables were summarised as means with standard deviations or medians with interquartile ranges, according to distribution. Categorical variables were summarised as frequencies and percentages. Cohort comparisons used the Student's t-test or Mann-Whitney U test for continuous data and the chi-square or Fisher's exact test for categorical data. Survival analyses were performed using Cox regression. A two-sided P < 0.05 indicated statistical significance. Image preprocessing, model training, statistical analysis and visualisation were performed in Python. DL models were developed with PyTorch, and standard Python machine-learning and survival-analysis libraries were used for performance evaluation. To evaluate sensitivity to potential informative censoring, stabilized inverse probability of censoring weights (IPCW) were estimated separately within each cohort using Cox models for potential early loss to follow-up. The censoring models included age, sex, tumour location, CEA, T stage, N stage, pathological type, differentiation grade, PNI, LVI, Tumor DL score, and VAT DL score. Weights were truncated at the 1st and 99th percentiles. The multivariable Cox model was refitted using robust variance estimates, model discrimination was recalculated, and risk-group survival was examined using IPCW-weighted Kaplan-Meier curves. Local recurrence and distant metastasis were analysed as mutually competing first events. Cumulative incidence functions were estimated using the Aalen-Johansen method and compared across training-derived risk groups using Gray's test. Associations of the z-standardized Tumor DL and VAT DL scores with each recurrence type were evaluated using cause-specific Cox models stratified by cohort and Fine-Gray subdistribution hazard models including cohort indicators; both models were adjusted for N stage and PNI. Lunn-McNeil data augmentation was used to test heterogeneity of the imaging-score associations between local recurrence and distant metastasis. An inference-only sensitivity analysis was performed to assess dependence on CT intensity windowing. The frozen Tumor DL and VAT DL checkpoints were applied without retraining after re-windowing the cached CT inputs to -150 to 250 HU and -250 to 350 HU, with -200 to 300 HU as the primary window. The corresponding final multimodal linear predictors were recalculated while holding the fitted Cox coefficients and clinical covariates fixed. Agreement with primary-window scores was quantified using ICC(2,1) and Spearman correlation. C-indices and 2-year cumulative/dynamic AUCs were recalculated in each cohort, with 95% confidence intervals obtained from 1,000 bootstrap resamples. Time-specific survival decision-curve analysis was performed at 12, 24, and 36 months. Absolute recurrence risks were derived using the Breslow baseline survival function estimated in the training cohort; model coefficients and baseline survival functions were locked during external evaluation. Net benefit was estimated using IPCW to account for right censoring, with the censoring distribution estimated separately within each evaluation cohort. Patients with recurrence by the prediction horizon were weighted by the inverse censoring probability immediately before their event time, and patients remaining recurrence-free beyond the horizon were weighted by the inverse censoring probability at that horizon. The postoperative multimodal model was compared with the clinicopathological-only model, a pTNM stage-only Cox model fitted in the training cohort, and treat-all and treat-none strategies. A threshold-probability range of 5%-30% was prespecified because it encompassed the observed 1- to 3-year recurrence risks and a plausible range for considering intensified postoperative surveillance or further treatment assessment; 1%-50% was examined in a sensitivity analysis. Net benefits and between-model differences at 10% and 20% thresholds were summarised with 95% confidence intervals from 1,000 patient-level bootstrap resamples.

Quantitative calibration of the postoperative multimodal model was assessed at 12, 24 and 36 months using calibration-in-the-large (ideal value, 0), calibration slope (ideal value, 1) and IPCW Brier scores. Calibration-in-the-large and slope were estimated using IPCW complementary-log-log calibration models, with 95% confidence intervals from 1,000 patient-level bootstrap resamples. Absolute risks were calculated from the training-cohort Breslow baseline cumulative hazard and locked final-model linear predictor; the external cohorts were evaluated without refitting, parameter updating or recalibration. Exploratory low-, intermediate- and high-risk groups were defined by the 33.3rd and 66.7th percentiles of the final-model risk score in the training cohort and applied unchanged to both external cohorts. Group-specific hazard ratios used the low-risk group as reference, and pairwise log-rank P values were adjusted within each cohort using the Holm method.

Because univariable screening may produce unstable variable selection, model robustness was additionally evaluated using a ridge-penalised Cox model that included all 14 candidate parameters: the Tumor DL and VAT DL scores and all 12 candidate clinicopathological variables, without univariable P-value filtering. The ridge penalty was selected exclusively in the training cohort using three repeats of 10-fold cross-validation based on validation partial log-likelihood. The nominal events-per-candidate-parameter ratio was reported for both the complete candidate set and the original postoperative multimodal model. Multicollinearity was assessed using variance inflation factors (VIFs), and proportional-hazards assumptions were evaluated for every candidate variable and imaging score using scaled Schoenfeld residual tests, together with a global test. Pathological N stage was coded as an ordinal variable (N0=0, N1=1 and N2=2), such that its hazard ratio represented a one-category increase; a two-degree indicator representation (N1 versus N0 and N2 versus N0) was evaluated by likelihood-ratio testing. Postoperative adjuvant therapy was not included in the primary prognostic model because it was administered after surgery and was strongly related to pathological risk, creating confounding by indication. The harmonised database recorded whether any postoperative adjuvant therapy was documented but did not consistently capture treatment-initiation dates, regimens, dose intensity, number of cycles or duration. Sensitivity analyses therefore examined whether the association between the standardised final risk score and RFS persisted after adjustment for treatment, pTNM stage and cohort; tested continuous risk-score-by-treatment and risk-score-by-stage interactions; and estimated score associations within treatment and pTNM subgroups. A propensity-score overlap-weighted analysis was also performed using age, sex, tumour location, CEA, T stage, N stage, pathological type, differentiation grade, PNI, LVI, the two imaging scores and cohort indicators. Covariate balance was assessed using absolute standardised mean differences, with 0.10 as the prespecified threshold. These analyses evaluated robustness to treatment allocation and were not interpreted as causal estimates of treatment benefit.

The clinicopathological-only model was evaluated using the same patients, endpoints, and cohort splits as the postoperative multimodal model. Patient-level paired bootstrap resampling with 1,000 replicates was used to estimate 95% confidence intervals for between-model differences in Harrell's C-index, 1-, 2-, and 3-year cumulative/dynamic AUCs, IPCW Brier scores, and the integrated Brier score (IBS) over 1-36 months. Improvement was defined as multimodal minus clinical-only for C-index and AUC and as clinical-only minus multimodal for Brier scores and IBS, so that positive values consistently favoured the postoperative multimodal model.

Cohort heterogeneity was evaluated using absolute standardised mean differences (SMDs), with the training cohort as reference; values of 0.10 and 0.20 were used as descriptive thresholds rather than significance tests. Major demographic, clinicopathological and treatment variables were assessed together with in-plane and through-plane voxel spacing. Protocol-level scanner models and typical acquisition and contrast parameters are listed in Table S1, and centre-specific enrolment periods are reported above. Because the analysis archive contained de-identified NIfTI volumes rather than source DICOM headers, patient-level scanner assignment, reconstruction kernel and exact contrast parameters could not be reconstructed. The locked postoperative multimodal score was evaluated without retraining according to centre, tumour location, pTNM stage, sex and adjuvant-treatment status using Harrell's C-index and 2-year cumulative/dynamic AUC with 1,000 patient-level bootstrap confidence intervals. Colon and rectal cancers were also evaluated separately within each centre. Score-by-subgroup interactions were tested in pooled Cox models with centre adjustment, except that the centre interaction was tested directly. Body mass index was not analysed because it was not consistently collected.

# Supplementary Results

## Patient characteristics, follow-up and recurrence patterns

Cohort heterogeneity was quantified using absolute standardised mean differences (SMDs). The largest clinical or pathological absolute SMD was 0.147 for external test cohort 1 and 0.141 for external test cohort 2, relative to the training cohort. Acquisition geometry was more heterogeneous: absolute SMDs for in-plane spacing were 0.432 and 0.380, while those for through-plane spacing were 1.855 and 1.074, respectively.

Despite this heterogeneity, the locked final model achieved C-indices of 0.843, 0.772 and 0.810 and 2-year area under the curve values of 0.870, 0.757 and 0.836 in the training cohort and external test cohorts 1 and 2, respectively. In pooled anatomical subgroups, C-indices were 0.810 for colon cancer and 0.824 for rectal cancer, with corresponding 2-year AUCs of 0.828 and 0.834. The score-by-location interaction was not significant (P=0.922). No interactions were identified by centre (P=0.515), sex (P=0.132) or adjuvant therapy (P=0.641), whereas the score association varied by pathological tumour-node-metastasis stage (P=0.002). Centre-specific colon and rectal cancer results are reported in Table S3 and Figure S1.

### Follow-up adequacy and censoring sensitivity

Reverse Kaplan–Meier median follow-up was 31 months (IQR, 19–40; observed range, 2–73) in the training cohort, 34 months (IQR, 21–48; range, 3–73) in external test cohort 1 and 36 months (IQR, 24–45; range, 2–64) in external test cohort 2. The respective numbers at risk at 12, 24 and 36 months were 610, 379 and 187; 378, 239 and 150; and 217, 161 and 90. By 36 months, cumulative first recurrence had occurred in 160, 91 and 56 patients, respectively. Complete time-specific event counts are provided in Table S4 and Figure S2.

Potential early loss to follow-up occurred in 200 of 705 patients (28.4%) in the training cohort, 118 of 425 (27.8%) in external test cohort 1 and 38 of 239 (15.9%) in external test cohort 2. Median follow-up before potential early loss was 15, 16 and 15 months, respectively.

After inverse probability of censoring weighting (IPCW), Tumor DL score (hazard ratio [HR], 2.026; 95% confidence interval [CI], 1.685–2.436; P < 0.001), VAT DL score (HR, 2.119; 95% CI, 1.825–2.459; P < 0.001) and N stage (HR, 1.520; 95% CI, 1.241–1.860; P < 0.001) remained independently associated with recurrence-free survival. The effect estimate for perineural invasion was similar, although its confidence interval included unity (HR, 1.478; 95% CI, 0.967–2.259; P = 0.071).

IPCW-adjusted C-indices were 0.844, 0.775 and 0.812 in the training cohort and external test cohorts 1 and 2, compared with unadjusted values of 0.843, 0.771 and 0.811, respectively. IPCW-weighted survival curves retained significant separation among all three risk groups in every cohort (all weighted Cox P < 0.001; Figure S3 and Table S5).

### Local and distant recurrence

Local recurrence occurred in 39, 13 and 11 patients in the training cohort and external test cohorts 1 and 2, respectively. Distant metastasis alone occurred in 121, 77 and 40 patients, while synchronous local and distant recurrence occurred in 13, 9 and 9 patients, respectively.

In pooled adjusted competing-risk analyses, both imaging scores were associated with local recurrence. For Tumor DL, the cause-specific HR was 1.731 (95% CI, 1.320–2.270) and the Fine–Gray subdistribution hazard ratio (sHR) was 1.510 (95% CI, 1.125–2.026). For VAT DL, the corresponding estimates were 2.337 (95% CI, 1.802–3.030) and 2.138 (95% CI, 1.587–2.879).

Both imaging scores were also associated with distant metastasis. For Tumor DL, the cause-specific HR was 2.035 (95% CI, 1.786–2.320) and the Fine–Gray sHR was 1.884 (95% CI, 1.638–2.166). For VAT DL, the corresponding estimates were 2.059 (95% CI, 1.809–2.344) and 1.648 (95% CI, 1.414–1.922). All imaging-score associations had P≤0.006.

Formal heterogeneity tests indicated no significant difference between local and distant recurrence associations for Tumor DL (P = 0.306) or VAT DL (P = 0.443). Final-model risk groups separated the cumulative incidence of both outcomes in the training cohort and external test cohort 2 (all Gray’s P < 0.001). In external test cohort 1, separation was significant for distant metastasis (P < 0.001) and borderline for local recurrence (P = 0.057). Excluding synchronous events produced similar estimates (Table S6 and Figure S4).

Organ-specific metastatic sites and deaths before documented recurrence could not be reliably enumerated because these fields were not harmonised across the participating retrospective databases.

## Reproducibility and sensitivity analyses

### Tumour-segmentation reproducibility

Tumour-mask overlap, boundary agreement and Tumor DL-score reproducibility in the 30-case observer study are presented in Figure S7. Final-model linear predictors derived from the original, interobserver and intraobserver tumour segmentations showed an intraclass correlation coefficient, ICC(2,1), of 0.953 (95% confidence interval [CI], 0.878–0.985). Agreement for the fixed training-derived low-, intermediate- and high-risk categories was 76.7% for both interobserver and intraobserver comparisons, with weighted kappa values of 0.734 and 0.761, respectively.

### Workflow audit and CT-window sensitivity

The preprocessing audit confirmed that deterministic 32-slice craniocaudal quantile sampling was applied consistently across all cohorts. Each patient had at least 57 valid VAT-containing slices (median, 450; range, 57–1,116), and none of the 1,369 patients required the predefined slice-duplication fallback.

Alternative CT intensity windows preserved risk-score ranking. Spearman correlation coefficients ranged from 0.9998 to 0.9999 for Tumor DL, 0.9718 to 0.9869 for VAT DL and 0.9897 to 0.9946 for the final fusion score. Compared with the primary window, ICC(2,1) values ranged from 0.9998 to 0.9999, 0.7773 to 0.8810 and 0.9145 to 0.9536, respectively.

Model discrimination was similarly stable. Final-model C-indices ranged from 0.839 to 0.844 in the training cohort, 0.767 to 0.772 in external test cohort 1 and 0.804 to 0.810 in external test cohort 2. Corresponding 2-year time-dependent area under the curve (AUC) values ranged from 0.866 to 0.871, 0.753 to 0.757 and 0.829 to 0.836, respectively (Table S7 and Figure S6).

### Physical-space and positional-encoding sensitivity analyses

No unreadable CT images were identified. Median in-plane spacing was 0.867 mm (interquartile range [IQR], 0.721–1.000) in the training cohort, 0.832 mm (IQR, 0.738–0.889) in external test cohort 1 and 1.000 mm (IQR, 0.736–1.000) in external test cohort 2. Median through-plane spacing was 1.000 mm in all cohorts, although the external ranges were wider, at 0.700–2.045 mm and 0.499–5.635 mm.

The fixed-physical-field Tumor sensitivity model achieved lower C-indices than the primary Tumor model in the training cohort (0.635 vs 0.775), external test cohort 1 (0.609 vs 0.681) and external test cohort 2 (0.603 vs 0.717). Positional encoding did not improve the VAT branch: C-indices were 0.701, 0.618 and 0.604, compared with 0.777, 0.671 and 0.708 for the primary VAT model. Corresponding 2-year AUCs and paired bootstrap differences are reported in Table S8 and Figure S8. These findings indicate that the primary models were dependent on their submitted preprocessing strategy and were not invariant to fixed physical-space resampling or explicit relative-position encoding.

### Repeated cross-validation and complete-risk-set analysis

Across three repeats of five-fold training-from-scratch cross-validation, the 15 fold-level C-indices averaged 0.556 (standard deviation [SD], 0.054; range, 0.426–0.639) for Tumor DL and 0.498 (SD, 0.064; range, 0.403–0.607) for VAT DL. Corresponding 2-year AUCs averaged 0.565 (SD, 0.063; range, 0.449–0.690) and 0.507 (SD, 0.073; range, 0.403–0.667), respectively.

Across the three repeats, mean pooled out-of-fold C-indices were 0.540 (SD, 0.013) for Tumor DL and 0.509 (SD, 0.015) for VAT DL, while mean pooled 2-year AUCs were 0.550 (SD, 0.026) and 0.514 (SD, 0.012). Averaging each patient’s three out-of-fold predictions yielded C-indices of 0.562 and 0.512 and 2-year AUCs of 0.578 and 0.522, respectively. These results indicate appreciable fold-to-fold variability and lower development-centre stability than suggested by the original single split (Table S9 and Figure S9).

Complete-risk-set Cox-head refitting produced external discrimination similar to the original mini-batch-trained heads. In external test cohort 1, C-indices were 0.678 versus 0.681 for Tumor DL and 0.671 versus 0.671 for VAT DL; corresponding 2-year AUCs were 0.662 versus 0.665 and 0.642 versus 0.648. In external test cohort 2, C-indices were 0.718 versus 0.717 and 0.708 versus 0.708, while 2-year AUCs were 0.746 versus 0.748 and 0.727 versus 0.725. All paired external differences were small, and their 95% bootstrap CIs included zero (Table S9 and Figure S9).

### Incremental value over the clinicopathological model

Absolute performance of the clinicopathological-only and postoperative multimodal models, together with paired differences in C-index, 1-, 2- and 3-year AUC, Brier score and IBS, is reported in Table S10 and Figure S10. Improvement was clear in the training cohort and external test cohort 2, whereas several differences in external test cohort 1 did not exclude zero.

## Additional model evaluation

### Calibration and decision-curve analysis

Visual calibration curves at 1, 2 and 3 years showed generally favourable agreement between predicted and observed recurrence risks. Quantitative calibration estimates are provided in Table S12. Calibration-in-the-large values in the training cohort were 0.001, −0.014 and 0.099 at 1, 2 and 3 years, with corresponding slopes of 0.983, 0.963 and 1.174.

In external test cohorts 1 and 2, 1-year calibration-in-the-large values were 0.428 (95% CI, 0.061–0.720) and 0.443 (95% CI, 0.008–0.795), respectively, indicating short-horizon underprediction. At 2 and 3 years, confidence intervals generally included zero, except for a modest positive 2-year intercept in external test cohort 1 (0.296; 95% CI, 0.010–0.556). Calibration slopes showed horizon-specific variation, most notably at 3 years in external test cohort 1 (1.441; 95% CI, 1.152–1.873). External predictions were evaluated without model updating or recalibration.

Inverse probability of censoring weighting-adjusted survival decision-curve analysis demonstrated positive net benefit for the postoperative multimodal model across the prespecified 5%–30% threshold range in all cohorts and at all three horizons (Figure 4). At thresholds of 10% and 20%, its point-estimated net benefit exceeded that of both the clinicopathological-only and pathological tumour-node-metastasis (pTNM) stage models in eight of nine cohort-horizon comparisons at each threshold.

The exceptions occurred in external test cohort 1, where the clinicopathological-only model showed slightly greater net benefit at 3 years using a 10% threshold and at 2 years using a 20% threshold. Thus, the incremental clinical utility of the multimodal model depended on the prediction horizon and decision threshold (Table S13 and Figure S12).

### Variable-selection and model-diagnostic sensitivity analyses

The training cohort contained 173 recurrence events, corresponding to 12.4 events per parameter for the complete 14-parameter candidate set and 19.2 events per parameter for the original nine-parameter postoperative multimodal model. Repeated 10-fold cross-validation selected a ridge penalty of 0.03.

The all-candidate ridge model achieved C-indices of 0.849, 0.787 and 0.812 in the training cohort and external test cohorts 1 and 2, compared with 0.843, 0.772 and 0.810 for the original model. Corresponding 2-year AUCs were 0.874, 0.782 and 0.837 for the ridge model and 0.870, 0.757 and 0.836 for the original model. Risk scores from the two models were highly correlated across cohorts (Spearman ρ=0.965–0.980).

The maximum variance inflation factor was 1.40. Neither the global scaled Schoenfeld residual test (P=0.874) nor any variable-specific test indicated violation of the proportional-hazards assumption. Treating N stage categorically did not significantly improve model fit over the ordinal representation (likelihood-ratio P=0.083), supporting interpretation of the reported N-stage HR as the effect of a one-category increase (Table S14 and Figure S13).

### Adjuvant-therapy sensitivity analyses

After adjustment for adjuvant therapy, pTNM stage and cohort, the final risk score remained strongly associated with recurrence-free survival (HR per one-standard-deviation increase, 3.607; 95% CI, 3.179–4.093; P < 0.001). The score-by-treatment interaction was not significant (interaction HR, 0.940; 95% CI, 0.672–1.315; P = 0.716), and the score remained prognostic in patients with and without documented adjuvant therapy.

The risk score was also significantly associated with recurrence-free survival within pTNM stages I, II and III, although its relative association decreased with advancing stage (score-by-stage interaction P=0.002). Because treatment allocation was strongly imbalanced, propensity-score overlap weighting was applied. The maximum absolute standardised mean difference decreased from 1.257 before weighting to 0.028 after weighting.

In the overlap-weighted model, the final risk score remained independently associated with recurrence-free survival (HR, 3.066; 95% CI, 2.089–4.501; P < 0.001). The score-by-treatment interaction remained non-significant (HR, 1.521; 95% CI, 0.983–2.353; P = 0.060) (Table S15 and Figure S14).

# Supplementary Tables

Table S1. CT scanning protocol for patients with CRC.

| Manufacture | SIEMENS | SIEMENS | SIEMENS | Philips | Philips | Toshiba |
| --- | --- | --- | --- | --- | --- | --- |
| CT scanner | CT256 | CT128 | CT128 | CT 256 | CT256 | CT320 |
| Scanner mode | SOMATOM Drive | SOMATOM Defifinition AS | SOMATOM Defifinition Edge | Brilliance iCT | IQon Spectral CT | Aquilion ONE |
| Tube voltage (kV) | 120 | 120 | 120 | 120 | 120 | 120 |
| Tube current (mA | 250 | 250 | 200 | 300-350 | 300 | 200 |
| Rotation time (s) | 0.5 | 0.5 | 0.5 | 0.5 | 0.4 | 0.5 |
| Collimation (mm) | 64*0.6 | 64*0.6 | 64*0.6 | 128*0.625 | 64*0.625 | 160*0.625 |
| Slice thickness (mm) | 1.25 | 2 | 1 | 2 | 1 | 3 |
| Matrix | 512 × 512 | 512 × 512 | 512 × 512 | 512 × 512 | 512 × 512 | 512 × 512 |
| Field of view (mm) | 350 | 350 | 350 | 350 | 350 | 350 |
| Kernel | standard | standard | B30f | Stardand | standard | Fc10 |

CT, computed tomography; CRC, colorectal cancer.

Table S2. Complete network architecture, training hyperparameters, and mathematical definitions.

| **Branch and stage** | **Output** | **Operation** | **Kernel/stride** | **Notes** |
| --- | --- | --- | --- | --- |
| Tumor 3D CNN: Input | 2 channels | Masked CT volume + binary mask | 80 x 80 x 80 voxels | Portal-venous CT; primary window -200 to 300 HU |
| Tumor 3D CNN: Stem | 10 | Conv3D + max pooling; BatchNorm + ReLU | 7x7x7/s2; pool 3x3x3/s2 | None |
| Tumor 3D CNN: Residual block 1 | 10 | Two Conv3D layers; BatchNorm + ReLU | 3x3x3/s1 | Identity shortcut |
| Tumor 3D CNN: Residual block 2 | 20 | Two Conv3D layers; BatchNorm + ReLU | 3x3x3; first layer s2 | 1x1x1/s2 projection shortcut |
| Tumor 3D CNN: Residual block 3 | 40 | Two Conv3D layers; BatchNorm + ReLU | 3x3x3; first layer s2 | 1x1x1/s2 projection shortcut |
| Tumor 3D CNN: Residual block 4 | 40 | Two Conv3D layers; BatchNorm + ReLU | 3x3x3/s1 | Identity shortcut |
| Tumor 3D CNN: Pooling | 40 | Adaptive global average pooling | 1x1x1 | None |
| Tumor 3D CNN: Feature head | 48 | Fully connected + dropout; ReLU | - | Dropout=0.55 |
| Tumor 3D CNN: Risk head | 1 | Linear | - | Continuous log-risk score |
| VAT attention MIL: Input | 2 channels/slice | 32 masked CT slices + binary masks | 32 x 160 x 160 | Deterministic 5th-95th percentile sampling |
| VAT attention MIL: Conv block 1 | 16 | Conv2D; BatchNorm + ReLU | 5x5/s2 | Shared across slices |
| VAT attention MIL: Conv block 2 | 32 | Conv2D; BatchNorm + ReLU | 3x3/s2 | Shared across slices |
| VAT attention MIL: Conv block 3 | 64 | Conv2D; BatchNorm + ReLU | 3x3/s2 | Shared across slices |
| VAT attention MIL: Conv block 4 | 64 | Conv2D; BatchNorm + ReLU | 3x3/s2 | Shared across slices |
| VAT attention MIL: Slice pooling | 64 | Adaptive global average pooling | 1x1 | None |
| VAT attention MIL: Slice feature | 64 | Fully connected; ReLU | - | Feature vector h_i |
| VAT attention MIL: Attention | 64 hidden; 1 weight/slice | Linear-tanh-linear + softmax; Tanh + softmax | - | Attention dimension=64 |
| VAT attention MIL: MIL aggregation | 64 | Weighted sum of slice features | - | z = sum_i a_i h_i |
| VAT attention MIL: Risk head | 1 | Dropout + linear | - | Dropout=0.35; continuous log-risk score |

**Table S3. Cohort heterogeneity, locked final-model subgroup performance and metadata availability.**

**A. Standardised mean differences**

| **Variable** | **External test 1 vs training** | **External test 2 vs training** |
| --- | --- | --- |
| Age, years | 0.007 | 0.083 |
| CEA >=5 ng/mL | 0.028 | 0.075 |
| Colon location | -0.007 | 0.002 |
| Drinking history | 0.127 | 0.043 |
| In-plane voxel spacing, mm | 0.432 | -0.380 |
| LVI present | -0.045 | 0.093 |
| Male sex | -0.066 | -0.141 |
| N1 | 0.108 | 0.065 |
| N2 | -0.079 | -0.040 |
| Other pathological type | -0.012 | -0.139 |
| PNI present | 0.072 | 0.097 |
| Poor differentiation | -0.029 | -0.006 |
| Postoperative adjuvant therapy | -0.147 | -0.059 |
| Smoking history | -0.046 | -0.036 |
| T3-4 | -0.130 | 0.007 |
| Through-plane voxel spacing, mm | 1.855 | 1.074 |
| pTNM stage II | -0.120 | -0.026 |
| pTNM stage III | 0.035 | 0.026 |

**B. Locked final-model subgroup performance**

| **Category** | **Subgroup** | **n** | **events** | **C-index (95% CI)** | **2-year AUC (95% CI)** |
| --- | --- | --- | --- | --- | --- |
| Centre | Training cohort | 705 | 173 | 0.843 (0.811-0.870) | 0.870 (0.826-0.910) |
| Centre | External test cohort 1 | 425 | 99 | 0.772 (0.721-0.823) | 0.757 (0.687-0.822) |
| Centre | External test cohort 2 | 239 | 60 | 0.810 (0.757-0.857) | 0.836 (0.765-0.899) |
| Tumour location | Colon | 725 | 176 | 0.810 (0.778-0.842) | 0.828 (0.788-0.869) |
| Tumour location | Rectum | 644 | 156 | 0.824 (0.791-0.857) | 0.834 (0.785-0.879) |
| pTNM stage | Stage I | 161 | 13 | 0.819 (0.693-0.940) | 0.813 (0.624-0.969) |
| pTNM stage | Stage II | 545 | 89 | 0.809 (0.755-0.863) | 0.803 (0.732-0.879) |
| pTNM stage | Stage III | 663 | 230 | 0.780 (0.750-0.807) | 0.810 (0.771-0.845) |
| Sex | Female | 582 | 132 | 0.804 (0.767-0.843) | 0.834 (0.785-0.883) |
| Sex | Male | 787 | 200 | 0.826 (0.795-0.855) | 0.831 (0.791-0.871) |
| Adjuvant therapy | No | 394 | 44 | 0.805 (0.728-0.872) | 0.796 (0.686-0.890) |
| Adjuvant therapy | Yes | 975 | 288 | 0.802 (0.776-0.826) | 0.825 (0.792-0.856) |
| Tumour location by centre | Training cohort: Colon | 374 | 90 | 0.848 (0.805-0.888) | 0.885 (0.829-0.932) |
| Tumour location by centre | Training cohort: Rectum | 331 | 83 | 0.838 (0.795-0.878) | 0.853 (0.785-0.915) |
| Tumour location by centre | External test cohort 1: Colon | 224 | 54 | 0.755 (0.679-0.828) | 0.744 (0.649-0.833) |
| Tumour location by centre | External test cohort 1: Rectum | 201 | 45 | 0.791 (0.722-0.866) | 0.768 (0.671-0.867) |
| Tumour location by centre | External test cohort 2: Colon | 127 | 32 | 0.782 (0.705-0.847) | 0.794 (0.684-0.896) |
| Tumour location by centre | External test cohort 2: Rectum | 112 | 28 | 0.838 (0.773-0.897) | 0.873 (0.792-0.940) |

**C. Score-by-subgroup interaction tests**

| **Modifier** | **Interaction P** | **Degrees of freedom** | **Interpretation** |
| --- | --- | --- | --- |
| Centre | 0.515 | 2 | No evidence of heterogeneity |
| Tumour location | 0.922 | 1 | No evidence of heterogeneity |
| pTNM stage | 0.002 | 2 | Evidence of heterogeneity |
| Sex | 0.132 | 1 | No evidence of heterogeneity |
| Adjuvant therapy | 0.641 | 1 | No evidence of heterogeneity |

**D. Acquisition and clinical metadata availability**

| **Requested field** | **Availability/handling** |
| --- | --- |
| Scanner manufacturer/model | Protocol-level scanner list available in Table S1; patient-level and centre-specific scanner counts were not retained after NIfTI conversion |
| Reconstruction kernel | Not retained after retrospective NIfTI conversion |
| Tube voltage/current | Protocol-level typical settings available in Table S1; patient-level settings were not retained consistently |
| Contrast dose, injection rate and exact delay | Protocol-level typical settings available in Appendix S1; patient-level values were not retained consistently |
| Centre-specific enrolment period | Centre 1: July 2018-June 2023; Centres 2 and 3: January 2019-September 2022 |
| Body mass index | Not collected consistently in the source clinical database |
| Available acquisition descriptors | In-plane and through-plane voxel spacing for all 1,369 cases |

**Table S4. Follow-up duration, numbers at risk, and recurrence-event composition by cohort.**

**A. Follow-up duration**

| **Cohort** | **n** | **Recurrence, n** | **Reverse-KM median (IQR), mo** | **Observed range, mo** |
| --- | --- | --- | --- | --- |
| Training cohort | 705 | 173 | 31 (19-40) | 2-73 |
| External test cohort 1 | 425 | 99 | 34 (21-48) | 3-73 |
| External test cohort 2 | 239 | 60 | 36 (24-45) | 2-64 |

**B. Numbers at risk and cumulative first recurrence events**

| **Cohort** | **Month** | **At risk** | **Any** | **Local** | **Distant** | **Synchronous** |
| --- | --- | --- | --- | --- | --- | --- |
| Training cohort | 12 | 610 | 70 | 24 | 38 | 8 |
| Training cohort | 24 | 379 | 129 | 37 | 80 | 12 |
| Training cohort | 36 | 187 | 160 | 39 | 108 | 13 |
| External test cohort 1 | 12 | 378 | 35 | 8 | 23 | 4 |
| External test cohort 1 | 24 | 239 | 70 | 13 | 49 | 8 |
| External test cohort 1 | 36 | 150 | 91 | 13 | 69 | 9 |
| External test cohort 2 | 12 | 217 | 27 | 7 | 17 | 3 |
| External test cohort 2 | 24 | 161 | 43 | 11 | 25 | 7 |
| External test cohort 2 | 36 | 90 | 56 | 11 | 36 | 9 |

Event counts are cumulative first documented recurrences by the stated horizon. Organ-specific distant-metastasis sites and deaths before recurrence were not uniformly available.

**Table S5. Potential early loss to follow-up and IPCW sensitivity analyses.**

**A. Follow-up completeness by cohort**

| **Cohort** | **Total** | **Recurrence** | **Potential early loss, n (%)** | **Follow-up before loss, median (IQR), months** |
| --- | --- | --- | --- | --- |
| Training cohort | 705 | 173 | 200 (28.4%) | 15.0 (12.0-19.0) |
| External test cohort 1 | 425 | 99 | 118 (27.8%) | 16.0 (12.0-20.0) |
| External test cohort 2 | 239 | 60 | 38 (15.9%) | 15.0 (12.2-20.0) |

**B. Original and IPCW-weighted multivariable Cox regression**

| **Variable** | **Original HR (95% CI)** | **Original P** | **IPCW HR (95% CI)** | **IPCW P** |
| --- | --- | --- | --- | --- |
| Tumor DL score (z-standardized) | 2.016 (1.694-2.399) | <0.001 | 2.026 (1.685-2.436) | <0.001 |
| VAT DL score (z-standardized) | 2.099 (1.808-2.436) | <0.001 | 2.119 (1.825-2.459) | <0.001 |
| CEA | 1.246 (0.905-1.714) | 0.177 | 1.250 (0.910-1.719) | 0.169 |
| N stage | 1.488 (1.217-1.820) | <0.001 | 1.520 (1.241-1.860) | <0.001 |
| Pathological type | 0.771 (0.513-1.158) | 0.211 | 0.787 (0.496-1.248) | 0.308 |
| Differentiation grade | 1.270 (0.823-1.960) | 0.280 | 1.224 (0.740-2.024) | 0.432 |
| PNI | 1.507 (1.015-2.238) | 0.042 | 1.478 (0.967-2.259) | 0.071 |
| LVI | 1.002 (0.700-1.433) | 0.993 | 0.998 (0.688-1.449) | 0.993 |
| T3-4 vs T1-2 | 1.018 (0.553-1.872) | 0.955 | 0.972 (0.565-1.670) | 0.917 |

**C. Model discrimination before and after IPCW adjustment**

| **Cohort** | **Original C-index** | **IPCW C-index, original score** | **IPCW C-index, refitted model** |
| --- | --- | --- | --- |
| Training cohort | 0.843 | 0.844 | 0.844 |
| External test cohort 1 | 0.771 | 0.775 | 0.777 |
| External test cohort 2 | 0.811 | 0.812 | 0.813 |

*Potential early loss to follow-up was conservatively defined as recurrence-free censoring before 24 months. Stabilized IPC weights were estimated separately within each cohort and truncated at the 1st and 99th percentiles. CI, confidence interval; HR, hazard ratio; IPCW, inverse probability of censoring weighting.*

**Table S6. Recurrence-pattern and competing-risk analyses.**

**A. Recurrence patterns by cohort**

| **Cohort** | **Total** | **No recurrence** | **Local recurrence** | **Distant metastasis** | **Synchronous local+distant** | **Any recurrence** |
| --- | --- | --- | --- | --- | --- | --- |
| Training cohort | 705 | 532 (75.5%) | 39 (5.5%) | 121 (17.2%) | 13 (1.8%) | 173 (24.5%) |
| External test cohort 1 | 425 | 326 (76.7%) | 13 (3.1%) | 77 (18.1%) | 9 (2.1%) | 99 (23.3%) |
| External test cohort 2 | 239 | 179 (74.9%) | 11 (4.6%) | 40 (16.7%) | 9 (3.8%) | 60 (25.1%) |
| Overall | 1369 | 1037 (75.7%) | 63 (4.6%) | 238 (17.4%) | 31 (2.3%) | 332 (24.3%) |

**B. Pooled adjusted cause-specific Cox and Fine-Gray models**

| **Endpoint** | **Predictor** | **Cause-specific HR (95% CI)** | **P** | **Fine-Gray sHR (95% CI)** | **P** |
| --- | --- | --- | --- | --- | --- |
| Local recurrence | Tumor DL score | 1.731 (1.320-2.270) | <0.001 | 1.510 (1.125-2.026) | 0.006 |
| Local recurrence | VAT DL score | 2.337 (1.802-3.030) | <0.001 | 2.138 (1.587-2.879) | <0.001 |
| Local recurrence | N stage | 1.556 (1.123-2.155) | 0.008 | 1.485 (1.066-2.068) | 0.019 |
| Local recurrence | PNI | 1.759 (1.018-3.040) | 0.043 | 1.617 (0.914-2.863) | 0.099 |
| Distant metastasis | Tumor DL score | 2.035 (1.786-2.320) | <0.001 | 1.884 (1.638-2.166) | <0.001 |
| Distant metastasis | VAT DL score | 2.059 (1.809-2.344) | <0.001 | 1.648 (1.414-1.922) | <0.001 |
| Distant metastasis | N stage | 1.520 (1.292-1.788) | <0.001 | 1.409 (1.193-1.663) | <0.001 |
| Distant metastasis | PNI | 1.699 (1.286-2.243) | <0.001 | 1.538 (1.120-2.111) | 0.008 |

**C. Heterogeneity tests and Gray tests**

| **Analysis** | **Variable/cohort** | **Endpoint or ratio of HRs (95% CI)** | **P** |
| --- | --- | --- | --- |
| Heterogeneity | Tumor DL score | 1.176 (0.862-1.605) | 0.306 |
| Heterogeneity | VAT DL score | 0.881 (0.637-1.218) | 0.443 |
| Gray test | Training cohort | Local recurrence | <0.001 |
| Gray test | Training cohort | Distant metastasis | <0.001 |
| Gray test | External test cohort 1 | Local recurrence | 0.057 |
| Gray test | External test cohort 1 | Distant metastasis | <0.001 |
| Gray test | External test cohort 2 | Local recurrence | <0.001 |
| Gray test | External test cohort 2 | Distant metastasis | <0.001 |

*Synchronous local and distant recurrence was classified as distant metastasis in the main analysis and excluded in sensitivity analysis. Models included z-standardized Tumor DL and VAT DL scores, N stage, and PNI. CI, confidence interval; CIF, cumulative incidence function; HR, hazard ratio; sHR, subdistribution hazard ratio. Full cohort-specific and sensitivity estimates are provided in the accompanying Table S6 workbook.*

**Table S7. Audit of the VAT preprocessing workflow and inference sensitivity to CT intensity window. The workbook reports the slice-sampling audit, score agreement with the primary window, and cohort-specific C-indices and 2-year cumulative/dynamic AUCs with bootstrap 95% confidence intervals.**

**Table S8. Image-preprocessing, positional-encoding and final risk-classification sensitivity analyses.**

**A. Original voxel-spacing distributions**

| **Cohort** | **Axis** | **Median (IQR), mm** | **Range, mm** |
| --- | --- | --- | --- |
| Training cohort | x | 0.867 (0.721-1.000) | 0.582-1.000 |
| Training cohort | y | 0.867 (0.721-1.000) | 0.582-1.000 |
| Training cohort | z | 1.000 (0.750-1.000) | 0.750-1.000 |
| External test cohort 1 | x | 0.832 (0.738-0.889) | 0.580-1.000 |
| External test cohort 1 | y | 0.832 (0.738-0.889) | 0.580-1.000 |
| External test cohort 1 | z | 1.000 (1.000-2.000) | 0.700-2.045 |
| External test cohort 2 | x | 1.000 (0.736-1.000) | 0.578-1.000 |
| External test cohort 2 | y | 1.000 (0.736-1.000) | 0.578-1.000 |
| External test cohort 2 | z | 1.000 (0.750-1.000) | 0.499-5.635 |

**B. Primary and sensitivity-model performance**

| **Model** | **Cohort** | **n** | **Events** | **C-index (95% CI)** | **2-year AUC (95% CI)** |
| --- | --- | --- | --- | --- | --- |
| Original Tumor | Training | 705 | 173 | 0.775 (0.739-0.810) | 0.803 (0.752-0.848) |
| Physical-space Tumor | Training | 705 | 173 | 0.635 (0.590-0.678) | 0.641 (0.584-0.697) |
| Original VAT | Training | 705 | 173 | 0.777 (0.738-0.814) | 0.800 (0.752-0.846) |
| Position-aware VAT | Training | 705 | 173 | 0.701 (0.655-0.744) | 0.721 (0.666-0.776) |
| Original Tumor | Test 1 | 425 | 99 | 0.681 (0.619-0.739) | 0.665 (0.583-0.735) |
| Physical-space Tumor | Test 1 | 425 | 99 | 0.609 (0.554-0.662) | 0.594 (0.516-0.666) |
| Original VAT | Test 1 | 425 | 99 | 0.671 (0.611-0.731) | 0.648 (0.569-0.723) |
| Position-aware VAT | Test 1 | 425 | 99 | 0.618 (0.561-0.672) | 0.640 (0.567-0.706) |
| Original Tumor | Test 2 | 239 | 60 | 0.717 (0.644-0.782) | 0.748 (0.661-0.829) |
| Physical-space Tumor | Test 2 | 239 | 60 | 0.603 (0.524-0.675) | 0.606 (0.508-0.701) |
| Original VAT | Test 2 | 239 | 60 | 0.708 (0.629-0.784) | 0.725 (0.627-0.815) |
| Position-aware VAT | Test 2 | 239 | 60 | 0.604 (0.520-0.679) | 0.603 (0.491-0.701) |

**C. Paired performance differences**

| **Comparison** | **Cohort** | **Delta C-index (95% CI)** | **Delta 2-year AUC (95% CI)** |
| --- | --- | --- | --- |
| Physical-space Tumor vs Original Tumor | Training | -0.140 (-0.185--0.098) | -0.162 (-0.217--0.108) |
| Position-aware VAT vs Original VAT | Training | -0.075 (-0.109--0.040) | -0.080 (-0.124--0.032) |
| Physical-space Tumor vs Original Tumor | Test 1 | -0.072 (-0.140--0.004) | -0.071 (-0.159-0.010) |
| Position-aware VAT vs Original VAT | Test 1 | -0.054 (-0.107--0.007) | -0.008 (-0.078-0.056) |
| Physical-space Tumor vs Original Tumor | Test 2 | -0.114 (-0.186--0.038) | -0.142 (-0.243--0.028) |
| Position-aware VAT vs Original VAT | Test 2 | -0.104 (-0.173--0.031) | -0.122 (-0.208--0.031) |

**D. Final-model risk-score and classification reproducibility**

| **Metric** | **Result** |
| --- | --- |
| Final linear-predictor ICC(2,1), three segmentations | 0.953 (0.878-0.985) |
| Final risk-group agreement, interobserver | 76.7% |
| Weighted kappa, interobserver | 0.734 |
| Final-score Spearman rho, interobserver | 0.921 |
| Final-score Spearman P, interobserver | <0.001 |
| Final risk-group agreement, intraobserver | 76.7% |
| Weighted kappa, intraobserver | 0.761 |
| Final-score Spearman rho, intraobserver | 0.910 |
| Final-score Spearman P, intraobserver | <0.001 |

**E. Preprocessing and software specification**

| **Item** | **Specification** |
| --- | --- |
| Primary Tumor preprocessing | No common-spacing resampling; 12-voxel array-index margin; bounding-box resize to 80x80x80; CT trilinear and mask nearest-neighbour interpolation |
| Physical-space Tumor sensitivity | 2.5-mm isotropic spacing; 96x96x96 fixed grid; 240-mm cubic field of view centred on tumour; CT linear and mask nearest-neighbour interpolation |
| Primary VAT sampling | 32 deterministic craniocaudal quantiles from the 5th to 95th percentiles of valid VAT-containing slices; no padding triggered in 1,369 patients |
| Position-aware VAT sensitivity | Normalised craniocaudal quantile coordinate embedded and added to each 64-dimensional slice representation before attention pooling |
| TotalSegmentator | Version 2.13.0; tissue_4_types task; GPU; default configuration; case-level visual QC; 0/1,369 voxel-level manual corrections |
| Blinding | Tumour readers and VAT quality-control reader were blinded to recurrence outcomes and model outputs |
| Primary cached-input augmentation | No random geometric augmentation was applied after cached tensors were loaded |

**Table S9. Deep-learning training procedure, repeated internal validation and complete-risk-set sensitivity analyses.**

**A. Original training configuration**

| **Parameter** | **Tumor 3D CNN** | **VAT attention MIL** |
| --- | --- | --- |
| Development cohort | 705 patients; 173 events | 705 patients; 173 events |
| Internal training subset | 564 patients; 138 events | 564 patients; 138 events |
| Internal validation subset | 141 patients; 35 events | 141 patients; 35 events |
| Split | 80:20; recurrence-status stratified | 80:20; recurrence-status stratified |
| Primary random seed | 2026 | 2026 |
| Optimiser | AdamW | AdamW |
| Learning rate | 8e-5, constant | 1e-4, constant |
| Learning-rate scheduler | None | None |
| Batch size | 6 | 8 |
| Maximum epochs | 25 | 35 |
| Epochs executed | 13 | 35 |
| Selected checkpoint epoch | 5 | 29 |
| Early stopping | Validation C-index patience 6 | Disabled |
| Dropout | 0.55 | 0.35 |
| Weight decay | 2e-4 | 5e-5 |
| Mixed precision | CUDA automatic mixed precision | CUDA automatic mixed precision |
| Effective online augmentation | None after cached-tensor loading | None after cached-tensor loading |
| Checkpoint rule | Highest validation C-index among checkpoints with development-cohort apparent C-index 0.70-0.80 | Highest validation C-index among checkpoints with development-cohort apparent C-index 0.70-0.80 |
| Transfer learning | No | No |
| External data in tuning | None | None |
| Primary Cox loss | Negative partial log-likelihood using risk sets constructed within each shuffled mini-batch | Negative partial log-likelihood using risk sets constructed within each shuffled mini-batch |

**B. Repeated three-times five-fold summary**

| **Model** | **Folds** | **Fold C-index, mean (SD)** | **Fold C-index, range** | **Fold 2-year AUC, mean (SD)** | **Fold 2-year AUC, range** | **Repeat-pooled C-index, mean (SD)** | **Repeat-pooled 2-year AUC, mean (SD)** | **Ensemble OOF C-index** | **Ensemble OOF 2-year AUC** |
| --- | --- | --- | --- | --- | --- | --- | --- | --- | --- |
| Tumor DL | 15 | 0.556 (0.054) | 0.426-0.639 | 0.565 (0.063) | 0.449-0.690 | 0.540 (0.013) | 0.550 (0.026) | 0.562 | 0.578 |
| VAT DL | 15 | 0.498 (0.064) | 0.403-0.607 | 0.507 (0.073) | 0.403-0.667 | 0.509 (0.015) | 0.514 (0.012) | 0.512 | 0.522 |

**C. Fold-level repeated cross-validation results**

| **model** | **repeat** | **fold** | **split seed** | **training seed** | **n** | **events** | **C index** | **AUC 2year** |
| --- | --- | --- | --- | --- | --- | --- | --- | --- |
| Tumor DL | 1 | 1 | 2026 | 2026 | 141 | 35 | 0.509 | 0.501 |
| Tumor DL | 1 | 2 | 2026 | 2027 | 141 | 35 | 0.606 | 0.604 |
| Tumor DL | 1 | 3 | 2026 | 2028 | 141 | 34 | 0.576 | 0.625 |
| Tumor DL | 1 | 4 | 2026 | 2029 | 141 | 34 | 0.518 | 0.570 |
| Tumor DL | 1 | 5 | 2026 | 2030 | 141 | 35 | 0.576 | 0.623 |
| VAT DL | 1 | 1 | 2026 | 2026 | 141 | 35 | 0.522 | 0.541 |
| VAT DL | 1 | 2 | 2026 | 2027 | 141 | 35 | 0.521 | 0.541 |
| VAT DL | 1 | 3 | 2026 | 2028 | 141 | 34 | 0.445 | 0.428 |
| VAT DL | 1 | 4 | 2026 | 2029 | 141 | 34 | 0.433 | 0.462 |
| VAT DL | 1 | 5 | 2026 | 2030 | 141 | 35 | 0.478 | 0.455 |
| Tumor DL | 2 | 1 | 2126 | 2126 | 141 | 35 | 0.516 | 0.520 |
| Tumor DL | 2 | 2 | 2126 | 2127 | 141 | 35 | 0.601 | 0.614 |
| Tumor DL | 2 | 3 | 2126 | 2128 | 141 | 34 | 0.573 | 0.501 |
| Tumor DL | 2 | 4 | 2126 | 2129 | 141 | 34 | 0.560 | 0.564 |
| Tumor DL | 2 | 5 | 2126 | 2130 | 141 | 35 | 0.426 | 0.449 |
| VAT DL | 2 | 1 | 2126 | 2126 | 141 | 35 | 0.438 | 0.451 |
| VAT DL | 2 | 2 | 2126 | 2127 | 141 | 35 | 0.558 | 0.541 |
| VAT DL | 2 | 3 | 2126 | 2128 | 141 | 34 | 0.607 | 0.667 |
| VAT DL | 2 | 4 | 2126 | 2129 | 141 | 34 | 0.571 | 0.575 |
| VAT DL | 2 | 5 | 2126 | 2130 | 141 | 35 | 0.505 | 0.515 |
| Tumor DL | 3 | 1 | 2226 | 2226 | 141 | 35 | 0.508 | 0.505 |
| Tumor DL | 3 | 2 | 2226 | 2227 | 141 | 35 | 0.563 | 0.591 |
| Tumor DL | 3 | 3 | 2226 | 2228 | 141 | 34 | 0.549 | 0.530 |
| Tumor DL | 3 | 4 | 2226 | 2229 | 141 | 34 | 0.621 | 0.588 |
| Tumor DL | 3 | 5 | 2226 | 2230 | 141 | 35 | 0.639 | 0.690 |
| VAT DL | 3 | 1 | 2226 | 2226 | 141 | 35 | 0.556 | 0.581 |
| VAT DL | 3 | 2 | 2226 | 2227 | 141 | 35 | 0.403 | 0.403 |
| VAT DL | 3 | 3 | 2226 | 2228 | 141 | 34 | 0.420 | 0.436 |
| VAT DL | 3 | 4 | 2226 | 2229 | 141 | 34 | 0.448 | 0.449 |
| VAT DL | 3 | 5 | 2226 | 2230 | 141 | 35 | 0.565 | 0.557 |

**D. Complete-risk-set Cox-head performance**

| **model** | **cohort** | **n** | **events** | **metric** | **estimate 95CI** |
| --- | --- | --- | --- | --- | --- |
| Original Tumor | Training cohort | 705 | 173 | C-index | 0.775 (0.739-0.810) |
| Full-risk-set Tumor Cox head | Training cohort | 705 | 173 | C-index | 0.780 (0.745-0.815) |
| Original VAT | Training cohort | 705 | 173 | C-index | 0.777 (0.736-0.818) |
| Full-risk-set VAT Cox head | Training cohort | 705 | 173 | C-index | 0.802 (0.764-0.838) |
| Original Tumor | Training cohort | 705 | 173 | 2-year AUC | 0.803 (0.752-0.848) |
| Full-risk-set Tumor Cox head | Training cohort | 705 | 173 | 2-year AUC | 0.808 (0.759-0.853) |
| Original VAT | Training cohort | 705 | 173 | 2-year AUC | 0.800 (0.751-0.848) |
| Full-risk-set VAT Cox head | Training cohort | 705 | 173 | 2-year AUC | 0.830 (0.784-0.873) |
| Original Tumor | External test cohort 1 | 425 | 99 | C-index | 0.681 (0.622-0.737) |
| Full-risk-set Tumor Cox head | External test cohort 1 | 425 | 99 | C-index | 0.678 (0.619-0.735) |
| Original VAT | External test cohort 1 | 425 | 99 | C-index | 0.671 (0.615-0.734) |
| Full-risk-set VAT Cox head | External test cohort 1 | 425 | 99 | C-index | 0.671 (0.614-0.728) |
| Original Tumor | External test cohort 1 | 425 | 99 | 2-year AUC | 0.665 (0.590-0.740) |
| Full-risk-set Tumor Cox head | External test cohort 1 | 425 | 99 | 2-year AUC | 0.662 (0.587-0.736) |
| Original VAT | External test cohort 1 | 425 | 99 | 2-year AUC | 0.648 (0.576-0.723) |
| Full-risk-set VAT Cox head | External test cohort 1 | 425 | 99 | 2-year AUC | 0.642 (0.568-0.718) |
| Original Tumor | External test cohort 2 | 239 | 60 | C-index | 0.717 (0.648-0.787) |
| Full-risk-set Tumor Cox head | External test cohort 2 | 239 | 60 | C-index | 0.718 (0.648-0.788) |
| Original VAT | External test cohort 2 | 239 | 60 | C-index | 0.708 (0.636-0.783) |
| Full-risk-set VAT Cox head | External test cohort 2 | 239 | 60 | C-index | 0.708 (0.637-0.784) |
| Original Tumor | External test cohort 2 | 239 | 60 | 2-year AUC | 0.748 (0.665-0.830) |
| Full-risk-set Tumor Cox head | External test cohort 2 | 239 | 60 | 2-year AUC | 0.746 (0.659-0.829) |
| Original VAT | External test cohort 2 | 239 | 60 | 2-year AUC | 0.725 (0.633-0.813) |
| Full-risk-set VAT Cox head | External test cohort 2 | 239 | 60 | 2-year AUC | 0.727 (0.638-0.814) |

**E. Paired complete-risk-set minus original differences**

| **branch** | **cohort** | **metric** | **difference 95CI** |
| --- | --- | --- | --- |
| Tumor | Training cohort | C-index | 0.005 (0.002-0.008) |
| VAT | Training cohort | C-index | 0.025 (0.016-0.034) |
| Tumor | Training cohort | 2-year AUC | 0.006 (0.002-0.009) |
| VAT | Training cohort | 2-year AUC | 0.030 (0.019-0.042) |
| Tumor | External test cohort 1 | C-index | -0.003 (-0.007-0.001) |
| VAT | External test cohort 1 | C-index | -0.001 (-0.011-0.009) |
| Tumor | External test cohort 1 | 2-year AUC | -0.003 (-0.008-0.002) |
| VAT | External test cohort 1 | 2-year AUC | -0.005 (-0.018-0.006) |
| Tumor | External test cohort 2 | C-index | 0.001 (-0.005-0.007) |
| VAT | External test cohort 2 | C-index | 0.000 (-0.015-0.015) |
| Tumor | External test cohort 2 | 2-year AUC | -0.002 (-0.011-0.006) |
| VAT | External test cohort 2 | 2-year AUC | 0.002 (-0.018-0.022) |

**F. Complete-risk-set Cox-head specification**

| **Branch** | **Retained features** | **Removed constant features** | **Selected ridge penalty** | **Risk sets** | **Ties** |
| --- | --- | --- | --- | --- | --- |
| Tumor DL | 38 | 10 | 3 | Complete 705-patient development-cohort risk sets | Breslow |
| VAT DL | 46 | 18 | 10 | Complete 705-patient development-cohort risk sets | Breslow |

**G. Cox-loss and risk-set definition**

| **Component** | **Definition** |
| --- | --- |
| Primary mini-batch loss | L_B = -[sum_i(delta_i)]^-1 sum_{i in B:delta_i=1} [r_i - log(sum_{j in B:t_j>=t_i} exp(r_j))] |
| Interpretation | Stochastic mini-batch approximation; risk sets were limited to patients present in each randomly shuffled batch. |
| Sensitivity analysis | Locked deep features were standardised and a ridge Cox head was fitted with complete development-cohort risk sets; constant feature channels were removed using the internal training subset only. |

**Table S10. Performance of the clinicopathological-only and postoperative multimodal models and paired incremental comparisons.**

**A. Discrimination**

| **Cohort** | **Model** | **C-index** | **1-y AUC** | **2-y AUC** | **3-y AUC** |
| --- | --- | --- | --- | --- | --- |
| Training cohort | Clinical-only | 0.707 (0.662-0.746) | 0.732 (0.661-0.797) | 0.701 (0.647-0.751) | 0.682 (0.621-0.740) |
| Training cohort | Postoperative multimodal | 0.843 (0.815-0.870) | 0.870 (0.827-0.911) | 0.870 (0.827-0.908) | 0.875 (0.832-0.917) |
| External test cohort 1 | Clinical-only | 0.709 (0.655-0.761) | 0.732 (0.650-0.821) | 0.746 (0.680-0.808) | 0.683 (0.613-0.756) |
| External test cohort 1 | Postoperative multimodal | 0.772 (0.716-0.824) | 0.808 (0.718-0.889) | 0.757 (0.686-0.822) | 0.827 (0.768-0.882) |
| External test cohort 2 | Clinical-only | 0.715 (0.652-0.777) | 0.760 (0.667-0.844) | 0.737 (0.650-0.812) | 0.716 (0.632-0.799) |
| External test cohort 2 | Postoperative multimodal | 0.810 (0.756-0.855) | 0.849 (0.773-0.912) | 0.836 (0.765-0.896) | 0.840 (0.769-0.899) |

**B. Prediction error**

| **Cohort** | **Model** | **1-y Brier** | **2-y Brier** | **3-y Brier** | **IBS 1-36 mo** |
| --- | --- | --- | --- | --- | --- |
| Training cohort | Clinical-only | 0.082 (0.066-0.099) | 0.150 (0.133-0.170) | 0.191 (0.167-0.216) | 0.111 (0.098-0.125) |
| Training cohort | Postoperative multimodal | 0.069 (0.055-0.084) | 0.103 (0.086-0.122) | 0.112 (0.091-0.133) | 0.080 (0.068-0.092) |
| External test cohort 1 | Clinical-only | 0.072 (0.054-0.091) | 0.134 (0.112-0.159) | 0.214 (0.181-0.248) | 0.101 (0.086-0.117) |
| External test cohort 1 | Postoperative multimodal | 0.066 (0.047-0.086) | 0.129 (0.103-0.159) | 0.156 (0.129-0.184) | 0.092 (0.075-0.109) |
| External test cohort 2 | Clinical-only | 0.090 (0.063-0.117) | 0.146 (0.117-0.179) | 0.222 (0.178-0.269) | 0.115 (0.093-0.136) |
| External test cohort 2 | Postoperative multimodal | 0.085 (0.058-0.112) | 0.130 (0.099-0.166) | 0.176 (0.136-0.221) | 0.099 (0.078-0.121) |

**C. Paired improvement: Training cohort**

| **Metric** | **Horizon, mo** | **Difference** | **Paired improvement (95% CI)** |
| --- | --- | --- | --- |
| C-index | Overall | Multimodal - clinical | 0.136 (0.092-0.178) |
| Time-dependent AUC | 12 | Multimodal - clinical | 0.138 (0.072-0.202) |
| Time-dependent AUC | 24 | Multimodal - clinical | 0.169 (0.112-0.223) |
| Time-dependent AUC | 36 | Multimodal - clinical | 0.193 (0.130-0.252) |
| Brier score | 12 | Clinical - multimodal | 0.013 (0.004-0.023) |
| Brier score | 24 | Clinical - multimodal | 0.047 (0.032-0.061) |
| Brier score | 36 | Clinical - multimodal | 0.080 (0.061-0.098) |
| Integrated Brier score | 1-36 | Clinical - multimodal | 0.032 (0.023-0.040) |

**D. Paired improvement: External test cohort 1**

| **Metric** | **Horizon, mo** | **Difference** | **Paired improvement (95% CI)** |
| --- | --- | --- | --- |
| C-index | Overall | Multimodal - clinical | 0.062 (-0.010-0.132) |
| Time-dependent AUC | 12 | Multimodal - clinical | 0.077 (-0.054-0.192) |
| Time-dependent AUC | 24 | Multimodal - clinical | 0.011 (-0.077-0.095) |
| Time-dependent AUC | 36 | Multimodal - clinical | 0.144 (0.057-0.226) |
| Brier score | 12 | Clinical - multimodal | 0.006 (-0.002-0.014) |
| Brier score | 24 | Clinical - multimodal | 0.005 (-0.012-0.022) |
| Brier score | 36 | Clinical - multimodal | 0.058 (0.032-0.084) |
| Integrated Brier score | 1-36 | Clinical - multimodal | 0.009 (-0.002-0.020) |

**E. Paired improvement: External test cohort 2**

| **Metric** | **Horizon, mo** | **Difference** | **Paired improvement (95% CI)** |
| --- | --- | --- | --- |
| C-index | Overall | Multimodal - clinical | 0.095 (0.023-0.168) |
| Time-dependent AUC | 12 | Multimodal - clinical | 0.089 (-0.010-0.194) |
| Time-dependent AUC | 24 | Multimodal - clinical | 0.099 (0.016-0.191) |
| Time-dependent AUC | 36 | Multimodal - clinical | 0.124 (0.032-0.220) |
| Brier score | 12 | Clinical - multimodal | 0.006 (-0.006-0.019) |
| Brier score | 24 | Clinical - multimodal | 0.016 (-0.007-0.040) |
| Brier score | 36 | Clinical - multimodal | 0.047 (0.005-0.086) |
| Integrated Brier score | 1-36 | Clinical - multimodal | 0.015 (0.001-0.031) |

Positive paired improvements favour the postoperative multimodal model. AUC denotes cumulative/dynamic time-dependent area under the curve; Brier scores use IPCW; IBS was integrated monthly from 1 to 36 months.

**Table S11. Time-dependent discrimination and prediction error of the imaging and postoperative multimodal models.**

**A. Cumulative/dynamic time-dependent AUC**

| **Cohort** | **Model** | **1 year** | **2 years** | **3 years** |
| --- | --- | --- | --- | --- |
| Training cohort | Tumor DL | 0.824 (0.776-0.867) | 0.803 (0.757-0.847) | 0.789 (0.738-0.841) |
| Training cohort | VAT DL | 0.787 (0.717-0.849) | 0.800 (0.748-0.847) | 0.829 (0.781-0.874) |
| Training cohort | Tumor+VAT | 0.860 (0.803-0.903) | 0.853 (0.806-0.894) | 0.863 (0.817-0.907) |
| Training cohort | Postoperative multimodal | 0.870 (0.825-0.910) | 0.870 (0.829-0.909) | 0.875 (0.828-0.917) |
| External test cohort 1 | Tumor DL | 0.681 (0.577-0.782) | 0.665 (0.587-0.740) | 0.749 (0.683-0.811) |
| External test cohort 1 | VAT DL | 0.671 (0.572-0.766) | 0.648 (0.572-0.718) | 0.713 (0.630-0.779) |
| External test cohort 1 | Tumor+VAT | 0.743 (0.644-0.826) | 0.712 (0.637-0.784) | 0.802 (0.739-0.861) |
| External test cohort 1 | Postoperative multimodal | 0.808 (0.716-0.886) | 0.757 (0.688-0.824) | 0.827 (0.768-0.885) |
| External test cohort 2 | Tumor DL | 0.756 (0.628-0.860) | 0.748 (0.663-0.826) | 0.698 (0.603-0.788) |
| External test cohort 2 | VAT DL | 0.687 (0.570-0.795) | 0.725 (0.630-0.804) | 0.773 (0.698-0.839) |
| External test cohort 2 | Tumor+VAT | 0.786 (0.697-0.863) | 0.807 (0.732-0.878) | 0.801 (0.723-0.871) |
| External test cohort 2 | Postoperative multimodal | 0.849 (0.780-0.913) | 0.836 (0.764-0.899) | 0.840 (0.769-0.907) |

**B. IPCW Brier score and integrated Brier score**

| **Cohort** | **Model** | **1 year** | **2 years** | **3 years** | **IBS 1-36 mo** |
| --- | --- | --- | --- | --- | --- |
| Training cohort | Tumor DL | 0.076 (0.062-0.091) | 0.130 (0.112-0.149) | 0.154 (0.130-0.177) | 0.098 (0.087-0.110) |
| Training cohort | VAT DL | 0.077 (0.062-0.093) | 0.125 (0.106-0.145) | 0.140 (0.117-0.163) | 0.095 (0.082-0.108) |
| Training cohort | Tumor+VAT | 0.070 (0.056-0.084) | 0.109 (0.091-0.128) | 0.119 (0.098-0.142) | 0.084 (0.072-0.096) |
| Training cohort | Postoperative multimodal | 0.069 (0.055-0.083) | 0.103 (0.085-0.120) | 0.112 (0.092-0.133) | 0.080 (0.068-0.091) |
| External test cohort 1 | Tumor DL | 0.072 (0.052-0.094) | 0.146 (0.119-0.175) | 0.194 (0.162-0.227) | 0.104 (0.088-0.123) |
| External test cohort 1 | VAT DL | 0.071 (0.053-0.093) | 0.146 (0.120-0.171) | 0.197 (0.168-0.224) | 0.105 (0.088-0.121) |
| External test cohort 1 | Tumor+VAT | 0.069 (0.048-0.089) | 0.138 (0.111-0.166) | 0.163 (0.135-0.192) | 0.097 (0.080-0.114) |
| External test cohort 1 | Postoperative multimodal | 0.066 (0.046-0.085) | 0.129 (0.103-0.157) | 0.156 (0.128-0.183) | 0.092 (0.075-0.110) |
| External test cohort 2 | Tumor DL | 0.088 (0.062-0.116) | 0.144 (0.113-0.179) | 0.223 (0.178-0.273) | 0.114 (0.093-0.136) |
| External test cohort 2 | VAT DL | 0.095 (0.066-0.125) | 0.146 (0.122-0.177) | 0.201 (0.166-0.236) | 0.112 (0.093-0.133) |
| External test cohort 2 | Tumor+VAT | 0.087 (0.058-0.114) | 0.134 (0.103-0.165) | 0.186 (0.143-0.229) | 0.103 (0.081-0.126) |
| External test cohort 2 | Postoperative multimodal | 0.085 (0.056-0.114) | 0.130 (0.096-0.162) | 0.176 (0.134-0.218) | 0.099 (0.077-0.121) |

Values are estimates with bootstrap 95% confidence intervals. AUC and Brier estimates used IPCW. IBS was calculated by integrating monthly Brier scores from 1 to 36 months. Lower Brier and IBS values indicate lower prediction error.

**Table S12. Quantitative calibration and exploratory risk stratification of the postoperative multimodal model.**

**A. Quantitative calibration and prediction error**

| **Cohort** | **Horizon** | **N** | **Events by horizon** | **Calibration-in-the-large (95% CI)** | **Calibration slope (95% CI)** | **IPCW Brier score (95% CI)** | **Valid bootstrap samples** |
| --- | --- | --- | --- | --- | --- | --- | --- |
| Training cohort | 1 year | 705 | 70 | 0.001 (-0.259-0.246) | 0.983 (0.824-1.193) | 0.069 (0.055-0.083) | 1000 |
| Training cohort | 2 year | 705 | 129 | -0.014 (-0.231-0.213) | 0.963 (0.809-1.249) | 0.103 (0.085-0.120) | 1000 |
| Training cohort | 3 year | 705 | 160 | 0.099 (-0.109-0.300) | 1.174 (0.948-1.533) | 0.112 (0.092-0.133) | 1000 |
| External test cohort 1 | 1 year | 425 | 35 | 0.428 (0.061-0.720) | 1.121 (0.795-1.642) | 0.066 (0.046-0.085) | 1000 |
| External test cohort 1 | 2 year | 425 | 70 | 0.296 (0.010-0.556) | 0.851 (0.600-1.273) | 0.129 (0.103-0.157) | 1000 |
| External test cohort 1 | 3 year | 425 | 91 | 0.092 (-0.162-0.328) | 1.441 (1.152-1.873) | 0.156 (0.128-0.183) | 1000 |
| External test cohort 2 | 1 year | 239 | 27 | 0.443 (0.008-0.795) | 1.264 (0.953-1.744) | 0.085 (0.056-0.114) | 1000 |
| External test cohort 2 | 2 year | 239 | 43 | 0.041 (-0.305-0.359) | 1.095 (0.834-1.665) | 0.130 (0.096-0.162) | 1000 |
| External test cohort 2 | 3 year | 239 | 56 | -0.204 (-0.549-0.157) | 1.043 (0.757-1.878) | 0.176 (0.134-0.218) | 1000 |

**B. Training-derived tertile cut-offs**

| **Boundary** | **Training quantile** | **Final risk-score cut-off** |
| --- | --- | --- |
| Low/intermediate boundary | 33.3rd percentile | 0.485813 |
| Intermediate/high boundary | 66.7th percentile | 1.560461 |

**C. Risk-group sample and event counts**

| **Cohort** | **Risk group** | **N** | **Events** |
| --- | --- | --- | --- |
| Training cohort | Low risk | 235 | 13 |
| Training cohort | Intermediate risk | 235 | 31 |
| Training cohort | High risk | 235 | 129 |
| External test cohort 1 | Low risk | 129 | 8 |
| External test cohort 1 | Intermediate risk | 201 | 37 |
| External test cohort 1 | High risk | 95 | 54 |
| External test cohort 2 | Low risk | 53 | 0 |
| External test cohort 2 | Intermediate risk | 102 | 16 |
| External test cohort 2 | High risk | 84 | 44 |

**D. Risk-group hazard ratios (low risk as reference)**

| **Cohort** | **Comparison** | **HR (95% CI)** | **P value** | **Estimation** |
| --- | --- | --- | --- | --- |
| Training cohort | Intermediate versus low | 2.77 (1.45-5.29) | 0.002 | Conventional Cox |
| Training cohort | High versus low | 16.01 (9.03-28.39) | <0.001 | Conventional Cox |
| External test cohort 1 | Intermediate versus low | 3.11 (1.45-6.69) | 0.004 | Conventional Cox |
| External test cohort 1 | High versus low | 12.55 (5.96-26.40) | <0.001 | Conventional Cox |
| External test cohort 2 | Intermediate versus low | 3.72 (1.21-11.50) | 0.022 | Ridge-penalised Cox (penalizer=0.01) |
| External test cohort 2 | High versus low | 18.14 (6.14-53.65) | <0.001 | Ridge-penalised Cox (penalizer=0.01) |

**E. Overall and pairwise log-rank tests**

| **Cohort** | **Comparison** | **Test statistic** | **Raw P value** | **Holm-adjusted P value** |
| --- | --- | --- | --- | --- |
| Training cohort | Overall three-group test | 221.384 | <0.001 | - |
| Training cohort | Intermediate risk versus Low risk | 11.24 | <0.001 | <0.001 |
| Training cohort | High risk versus Low risk | 154.669 | <0.001 | <0.001 |
| Training cohort | High risk versus Intermediate risk | 96.192 | <0.001 | <0.001 |
| External test cohort 1 | Overall three-group test | 94.748 | <0.001 | - |
| External test cohort 1 | Intermediate risk versus Low risk | 9.514 | 0.002 | 0.002 |
| External test cohort 1 | High risk versus Low risk | 71.733 | <0.001 | <0.001 |
| External test cohort 1 | High risk versus Intermediate risk | 49.685 | <0.001 | <0.001 |
| External test cohort 2 | Overall three-group test | 68.195 | <0.001 | - |
| External test cohort 2 | Intermediate risk versus Low risk | 9.417 | 0.002 | 0.002 |
| External test cohort 2 | High risk versus Low risk | 41.497 | <0.001 | <0.001 |
| External test cohort 2 | High risk versus Intermediate risk | 34.745 | <0.001 | <0.001 |

**Table S13. IPCW-adjusted survival decision-curve net benefit at representative threshold probabilities.**

| **Cohort** | **Horizon** | **Threshold** | **Multimodal NB** | **Clinical NB** | **pTNM NB** | **Difference vs clinical** | **Difference vs pTNM** |
| --- | --- | --- | --- | --- | --- | --- | --- |
| Training cohort | 1 year | 10% | 0.060 (0.039 to 0.081) | 0.039 (0.020 to 0.060) | 0.025 (0.004 to 0.046) | 0.021 (0.008 to 0.036) | 0.035 (0.022 to 0.048) |
| Training cohort | 1 year | 20% | 0.039 (0.021 to 0.059) | 0.023 (0.006 to 0.040) | 0.000 (0.000 to 0.000) | 0.017 (0.001 to 0.033) | 0.039 (0.021 to 0.059) |
| Training cohort | 2 year | 10% | 0.153 (0.121 to 0.186) | 0.121 (0.087 to 0.157) | 0.123 (0.089 to 0.158) | 0.032 (0.019 to 0.044) | 0.030 (0.018 to 0.045) |
| Training cohort | 2 year | 20% | 0.129 (0.099 to 0.164) | 0.059 (0.032 to 0.088) | 0.057 (0.026 to 0.091) | 0.070 (0.046 to 0.097) | 0.072 (0.051 to 0.092) |
| Training cohort | 3 year | 10% | 0.227 (0.191 to 0.271) | 0.213 (0.172 to 0.260) | 0.211 (0.169 to 0.258) | 0.014 (-0.007 to 0.030) | 0.015 (-0.006 to 0.033) |
| Training cohort | 3 year | 20% | 0.203 (0.159 to 0.247) | 0.131 (0.085 to 0.177) | 0.125 (0.073 to 0.177) | 0.072 (0.042 to 0.099) | 0.078 (0.048 to 0.102) |
| External test cohort 1 | 1 year | 10% | 0.039 (0.017 to 0.060) | 0.023 (0.002 to 0.046) | 0.013 (-0.008 to 0.037) | 0.016 (-0.003 to 0.033) | 0.025 (0.005 to 0.044) |
| External test cohort 1 | 1 year | 20% | 0.019 (0.005 to 0.036) | 0.010 (-0.008 to 0.029) | 0.000 (0.000 to 0.000) | 0.009 (-0.013 to 0.031) | 0.019 (0.005 to 0.036) |
| External test cohort 1 | 2 year | 10% | 0.103 (0.064 to 0.140) | 0.098 (0.055 to 0.142) | 0.099 (0.057 to 0.143) | 0.005 (-0.019 to 0.027) | 0.004 (-0.021 to 0.026) |
| External test cohort 1 | 2 year | 20% | 0.059 (0.028 to 0.092) | 0.074 (0.037 to 0.117) | 0.049 (0.009 to 0.095) | -0.016 (-0.049 to 0.014) | 0.010 (-0.025 to 0.042) |
| External test cohort 1 | 3 year | 10% | 0.198 (0.146 to 0.253) | 0.206 (0.150 to 0.263) | 0.195 (0.140 to 0.251) | -0.008 (-0.026 to 0.008) | 0.002 (-0.018 to 0.024) |
| External test cohort 1 | 3 year | 20% | 0.171 (0.116 to 0.226) | 0.131 (0.075 to 0.190) | 0.115 (0.054 to 0.180) | 0.040 (0.004 to 0.078) | 0.056 (0.022 to 0.089) |
| External test cohort 2 | 1 year | 10% | 0.057 (0.026 to 0.092) | 0.048 (0.011 to 0.085) | 0.043 (0.005 to 0.084) | 0.009 (-0.024 to 0.040) | 0.014 (-0.020 to 0.046) |
| External test cohort 2 | 1 year | 20% | 0.029 (0.007 to 0.056) | 0.021 (-0.005 to 0.050) | 0.000 (0.000 to 0.000) | 0.008 (-0.021 to 0.038) | 0.029 (0.007 to 0.056) |
| External test cohort 2 | 2 year | 10% | 0.131 (0.078 to 0.187) | 0.113 (0.058 to 0.172) | 0.107 (0.053 to 0.165) | 0.018 (-0.001 to 0.035) | 0.025 (0.009 to 0.038) |
| External test cohort 2 | 2 year | 20% | 0.096 (0.051 to 0.144) | 0.061 (0.019 to 0.111) | 0.036 (-0.010 to 0.089) | 0.035 (-0.001 to 0.070) | 0.060 (0.022 to 0.098) |
| External test cohort 2 | 3 year | 10% | 0.216 (0.146 to 0.285) | 0.199 (0.126 to 0.268) | 0.196 (0.125 to 0.265) | 0.017 (0.009 to 0.026) | 0.020 (0.009 to 0.033) |
| External test cohort 2 | 3 year | 20% | 0.163 (0.095 to 0.231) | 0.138 (0.069 to 0.206) | 0.108 (0.035 to 0.191) | 0.025 (-0.016 to 0.069) | 0.055 (0.013 to 0.089) |

Values are net benefit (95% CI) estimated using 1,000 patient-level bootstrap resamples. Differences were calculated as postoperative multimodal minus the named comparator; positive values favour the postoperative multimodal model. The pTNM stage-only Cox model was fitted in the training cohort and applied without refitting to both external cohorts.

**Table S14. Variable-selection, penalised-Cox and model-diagnostic sensitivity analyses.**

**A. Events per candidate parameter**

| **Model** | **Events** | **Parameters** | **Events per parameter** |
| --- | --- | --- | --- |
| All candidate parameters | 173 | 14 | 12.4 |
| Original postoperative multimodal model | 173 | 9 | 19.2 |

**B. Performance of the original and ridge-penalised models**

| **Cohort** | **Model** | **C-index (95% CI)** | **2-year AUC (95% CI)** | **IBS (95% CI)** |
| --- | --- | --- | --- | --- |
| Training cohort | Original univariable-screened Cox | 0.843 (0.811-0.870) | 0.870 (0.826-0.910) | 0.080 (0.069-0.092) |
| Training cohort | All-candidate ridge Cox | 0.849 (0.820-0.875) | 0.874 (0.831-0.913) | 0.079 (0.068-0.090) |
| External test cohort 1 | Original univariable-screened Cox | 0.772 (0.719-0.822) | 0.757 (0.686-0.821) | 0.092 (0.075-0.108) |
| External test cohort 1 | All-candidate ridge Cox | 0.787 (0.737-0.834) | 0.782 (0.717-0.842) | 0.089 (0.074-0.104) |
| External test cohort 2 | Original univariable-screened Cox | 0.810 (0.756-0.856) | 0.836 (0.766-0.897) | 0.099 (0.078-0.123) |
| External test cohort 2 | All-candidate ridge Cox | 0.812 (0.762-0.858) | 0.837 (0.771-0.900) | 0.099 (0.079-0.121) |

**C. Risk-score agreement**

| **Cohort** | **n** | **Spearman rho** |
| --- | --- | --- |
| Training cohort | 705 | 0.980 |
| External test cohort 1 | 425 | 0.965 |
| External test cohort 2 | 239 | 0.967 |

**D. All-candidate ridge-Cox coefficients**

| **Variable** | **HR (95% CI)** | **P** |
| --- | --- | --- |
| Tumor DL score | 1.875 (1.598-2.201) | <0.001 |
| VAT DL score | 2.009 (1.736-2.326) | <0.001 |
| Tumour location | 0.910 (0.677-1.224) | 0.534 |
| Sex | 0.670 (0.479-0.937) | 0.019 |
| Age | 1.102 (0.948-1.280) | 0.205 |
| Smoking history | 0.973 (0.670-1.412) | 0.885 |
| Drinking history | 1.091 (0.705-1.690) | 0.695 |
| CEA | 1.219 (0.901-1.649) | 0.200 |
| N stage (ordinal) | 1.464 (1.216-1.763) | <0.001 |
| Pathological type | 0.897 (0.607-1.325) | 0.585 |
| Differentiation grade | 1.086 (0.721-1.635) | 0.694 |
| PNI | 1.408 (0.975-2.033) | 0.068 |
| LVI | 1.085 (0.779-1.511) | 0.628 |
| T3-4 vs T1-2 | 1.010 (0.578-1.765) | 0.972 |

**E. Multicollinearity and proportional-hazards diagnostics**

| **Variable** | **VIF** | **PH chi-square** | **PH P** |
| --- | --- | --- | --- |
| Tumor DL score | 1.20 | 1.139 | 0.286 |
| VAT DL score | 1.20 | 3.088 | 0.079 |
| Tumour location | 1.11 | 0.075 | 0.784 |
| Sex | 1.34 | 0.000 | 0.993 |
| Age | 1.08 | 0.010 | 0.920 |
| Smoking history | 1.40 | 0.064 | 0.801 |
| Drinking history | 1.23 | 0.070 | 0.791 |
| CEA | 1.05 | 0.000 | 0.998 |
| N stage (ordinal) | 1.30 | 0.323 | 0.570 |
| Pathological type | 1.06 | 0.299 | 0.585 |
| Differentiation grade | 1.08 | 0.526 | 0.468 |
| PNI | 1.32 | 0.632 | 0.427 |
| LVI | 1.34 | 0.160 | 0.689 |
| T3-4 vs T1-2 | 1.18 | 1.903 | 0.168 |

**F. N-stage parameterisation sensitivity**

| **Parameterisation** | **Variable** | **HR (95% CI)** | **P** |
| --- | --- | --- | --- |
| Ordinal N0-N1-N2 | N | 1.488 (1.217-1.820) | <0.001 |
| Categorical N stage | N1_vs_N0 | 2.032 (1.356-3.044) | <0.001 |
| Categorical N stage | N2_vs_N0 | 2.230 (1.473-3.376) | <0.001 |

**Table S15. Postoperative adjuvant-therapy sensitivity and subgroup analyses.**

**A. Treatment distribution by cohort and pTNM stage**

| **Cohort** | **pTNM stage** | **Adjuvant therapy** | **n (events)** |
| --- | --- | --- | --- |
| Training cohort | 1 | No | 65 (7) |
| Training cohort | 1 | Yes | 9 (1) |
| Training cohort | 2 | No | 95 (10) |
| Training cohort | 2 | Yes | 200 (33) |
| Training cohort | 3 | No | 25 (9) |
| Training cohort | 3 | Yes | 311 (113) |
| External test cohort 1 | 1 | No | 59 (3) |
| External test cohort 1 | 1 | Yes | 3 (1) |
| External test cohort 1 | 2 | No | 74 (8) |
| External test cohort 1 | 2 | Yes | 79 (21) |
| External test cohort 1 | 3 | No | 7 (0) |
| External test cohort 1 | 3 | Yes | 203 (66) |
| External test cohort 2 | 1 | No | 21 (0) |
| External test cohort 2 | 1 | Yes | 4 (1) |
| External test cohort 2 | 2 | No | 41 (3) |
| External test cohort 2 | 2 | Yes | 56 (14) |
| External test cohort 2 | 3 | No | 7 (4) |
| External test cohort 2 | 3 | Yes | 110 (38) |

**B. Treatment-adjusted and interaction analyses**

| **Model** | **Variable** | **HR (95% CI)** | **P** |
| --- | --- | --- | --- |
| Stage- and cohort-adjusted sensitivity model | Original_final_score_z | 3.607 (3.179-4.093) | <0.001 |
| Stage- and cohort-adjusted sensitivity model | Adjuvant_therapy | 1.571 (1.094-2.255) | 0.014 |
| Continuous score-treatment interaction | Original_final_score_z | 3.813 (2.757-5.273) | <0.001 |
| Continuous score-treatment interaction | Adjuvant_therapy | 1.626 (1.081-2.446) | 0.020 |
| Continuous score-treatment interaction | score_x_therapy | 0.940 (0.672-1.315) | 0.716 |
| Continuous score-stage interaction | Original_final_score_z | 10.626 (5.364-21.047) | <0.001 |
| Continuous score-stage interaction | score_x_stage | 0.676 (0.530-0.862) | 0.002 |

**C. Final-score associations in treatment and stage subgroups**

| **Subgroup** | **n** | **Events** | **HR per 1-SD score (95% CI)** | **P** |
| --- | --- | --- | --- | --- |
| Adjuvant therapy: No | 394 | 44 | 3.324 (2.300-4.804) | <0.001 |
| Adjuvant therapy: Yes | 975 | 288 | 3.647 (3.186-4.176) | <0.001 |
| pTNM stage I | 161 | 13 | 8.533 (3.497-20.821) | <0.001 |
| pTNM stage II | 545 | 89 | 5.172 (3.846-6.957) | <0.001 |
| pTNM stage III | 663 | 230 | 3.182 (2.752-3.679) | <0.001 |

**D. Covariate balance before and after overlap weighting**

| **Variable** | **Absolute SMD before** | **Absolute SMD after** |
| --- | --- | --- |
| Age | 0.097 | 0.002 |
| Sex | 0.098 | 0.000 |
| Tumour location | 0.278 | 0.001 |
| CEA | 0.165 | 0.002 |
| T3-4 vs T1-2 | 0.874 | 0.004 |
| N stage (ordinal) | 1.257 | 0.025 |
| Pathological type | 0.166 | 0.003 |
| Differentiation grade | 0.166 | 0.007 |
| PNI | 0.768 | 0.028 |
| LVI | 0.913 | 0.022 |
| Tumor DL score | 0.177 | 0.002 |
| VAT DL score | 0.223 | 0.003 |
| External test cohort 1 | 0.135 | 0.001 |
| External test cohort 2 | 0.002 | 0.001 |

**E. Overlap-weighted sensitivity model**

| **Variable** | **HR (95% CI)** | **P** |
| --- | --- | --- |
| Original_final_score_z | 3.066 (2.089-4.501) | <0.001 |
| Adjuvant_therapy | 1.032 (0.654-1.627) | 0.893 |
| score_x_therapy | 1.521 (0.983-2.353) | 0.060 |

Table S16. Ablation analysis of imaging and clinical model components.

**A. Harrell's C-index**

| **Model** | **Training** | **External test 1** | **External test 2** |
| --- | --- | --- | --- |
| Clinical | 0.707 (0.662-0.746) | 0.709 (0.657-0.757) | 0.715 (0.648-0.772) |
| Tumor DL | 0.775 (0.743-0.807) | 0.681 (0.620-0.738) | 0.717 (0.645-0.790) |
| VAT DL | 0.777 (0.736-0.817) | 0.671 (0.611-0.732) | 0.708 (0.629-0.781) |
| Tumor+VAT | 0.828 (0.797-0.858) | 0.734 (0.678-0.793) | 0.771 (0.713-0.824) |
| Tumor+Clinical | 0.809 (0.779-0.837) | 0.742 (0.690-0.794) | 0.762 (0.696-0.823) |
| VAT+Clinical | 0.808 (0.773-0.842) | 0.763 (0.714-0.813) | 0.796 (0.738-0.851) |
| Tumor+VAT+Clinical | 0.843 (0.815-0.870) | 0.772 (0.719-0.825) | 0.810 (0.762-0.855) |

**B. 2-year cumulative/dynamic AUC**

| **Model** | **Training** | **External test 1** | **External test 2** |
| --- | --- | --- | --- |
| Clinical | 0.701 (0.647-0.751) | 0.746 (0.676-0.808) | 0.737 (0.650-0.811) |
| Tumor DL | 0.803 (0.757-0.847) | 0.665 (0.587-0.735) | 0.748 (0.663-0.830) |
| VAT DL | 0.800 (0.751-0.847) | 0.648 (0.570-0.719) | 0.725 (0.630-0.815) |
| Tumor+VAT | 0.853 (0.807-0.892) | 0.712 (0.642-0.780) | 0.807 (0.735-0.868) |
| Tumor+Clinical | 0.828 (0.782-0.869) | 0.739 (0.672-0.801) | 0.791 (0.707-0.870) |
| VAT+Clinical | 0.830 (0.786-0.870) | 0.773 (0.706-0.833) | 0.812 (0.728-0.879) |
| Tumor+VAT+Clinical | 0.870 (0.827-0.908) | 0.757 (0.691-0.822) | 0.836 (0.767-0.893) |

Values are estimates with bootstrap 95% confidence intervals. Full paired differences and Cox coefficients are provided in the accompanying Table S16 workbook.

# Supplementary Figures

Figure S1. Cohort heterogeneity and subgroup transportability. (A) Absolute standardised mean differences for major demographic, clinicopathological, treatment and voxel-spacing variables, using the training cohort as reference; dashed and dotted lines indicate descriptive thresholds of 0.10 and 0.20. (B) Harrell's C-index and 2-year cumulative/dynamic AUC of the locked postoperative multimodal model according to centre, tumour location, pTNM stage, sex and adjuvant-treatment status. Points show estimates and error bars show percentile 95% confidence intervals from 1,000 patient-level bootstrap resamples. Interaction P values were obtained from score-by-subgroup Cox models; absence of a significant interaction was not interpreted as equivalence.

Figure S2. Follow-up adequacy and recurrence-event composition. (A) Numbers of patients at risk at 1, 2, and 3 years in the training cohort and two external test cohorts; panel headings report reverse Kaplan-Meier median follow-up, interquartile range, and observed range. (B) Cumulative first documented recurrence events at the same horizons, classified as local only, distant only, or synchronous local and distant recurrence. Organ-specific distant-metastasis sites and deaths before recurrence were not uniformly available.

Figure S3. IPCW-weighted recurrence-free survival curves for the final multimodal model. Patients were classified into low-, intermediate-, and high-risk groups using tertile cut-offs derived from the training cohort. Stabilized inverse probability of censoring weights were estimated separately within each cohort using baseline clinicopathologic variables and imaging scores and truncated at the 1st and 99th percentiles. Vertical tick marks indicate censored observations. Risk-group separation remained significant in the training cohort and both external test cohorts (all IPCW-weighted Cox P < 0.001).

Figure S4. Cumulative incidence of local recurrence and distant metastasis according to the final multimodal model. Patients were classified into low-, intermediate-, and high-risk groups using tertile cut-offs derived from the training cohort. Local recurrence and distant metastasis were treated as mutually competing first events, with synchronous local and distant recurrence classified as distant metastasis in the primary analysis. Cumulative incidence functions were estimated using the Aalen-Johansen method and compared using Gray's test.

**Figure S5.** Kaplan-Meier recurrence-free survival curves for the tumour and VAT single-modality DL models. Patients were classified into high- and low-risk groups using cut-offs derived from the training cohort. Curves are shown for the training cohort and two external test cohorts. Vertical tick marks indicate censored observations, and numbers at risk are provided at 0, 12, 24, 36, 48 and 60 months. Both tumour and VAT DL scores stratified recurrence-free survival across all cohorts (all log-rank P < 0.001).

 Figure S6. Sensitivity of model performance to CT intensity window. C-indices (A) and 2-year cumulative/dynamic AUCs (B) are shown for the Tumor DL, VAT DL, and final multimodal fusion models in the training cohort and two external test cohorts after inference with -150 to 250 HU, the primary -200 to 300 HU window, and -250 to 350 HU. Model checkpoints were frozen and no retraining was performed. Points indicate estimates and error bars indicate 95% confidence intervals from 1,000 bootstrap resamples.

Figure S7. Reproducibility of manual tumour segmentation and the Tumor DL score. (A) Dice similarity coefficients for interobserver and intraobserver tumour-mask agreement in 30 randomly selected patients. (B) HD95 values for interobserver and intraobserver comparisons. (C) Agreement between Tumor DL scores generated from the original and repeated tumour segmentations. The dashed identity line represents perfect agreement. (D) ICCs with 95% confidence intervals for tumour volume and the Tumor DL score. ICCs were calculated using a two-way random-effects model with absolute agreement [ICC(2,1)].

 Figure S8. Image-preprocessing, positional-encoding and final risk-classification sensitivity analyses. (A) Original CT voxel-spacing distributions in the training cohort and two external test cohorts. (B) Harrell's C-index and 2-year cumulative/dynamic AUC of the primary Tumor model and the strict physical-space Tumor sensitivity model; points show estimates and error bars show 95% confidence intervals from 1,000 patient-level bootstrap resamples. (C) Corresponding comparison of the primary VAT model and position-aware VAT sensitivity model. (D) Final-model low-, intermediate- and high-risk classification agreement when the original tumour segmentation was replaced by the interobserver or intraobserver repeated segmentation in 30 patients.

Figure S9. Deep-learning training procedure, internal stability and risk-set sensitivity analyses. (A) Patient and recurrence-event distributions in the fixed internal training and validation subsets. (B) Original Tumor DL and VAT DL training Cox-loss and validation-C-index trajectories; dashed lines denote retained checkpoint epochs. (C) Fold-level C-indices and 2-year cumulative/dynamic AUCs from three repeats of five-fold from-scratch internal validation; points denote individual folds. (D) Original mini-batch-trained imaging models and complete-risk-set Cox-head sensitivity models in the training cohort and two external test cohorts. Error bars in panel D show 95% confidence intervals from 1,000 patient-level bootstrap resamples.

Figure S10. Paired incremental performance of the postoperative multimodal model over the clinicopathological-only model. Differences in C-index and time-dependent AUC were calculated as multimodal minus clinical-only; the IBS difference was calculated as clinical-only minus multimodal, so positive values consistently favour the postoperative multimodal model. Points show paired differences and error bars show percentile 95% confidence intervals from 1,000 patient-level bootstrap resamples.

Figure S11. Time-dependent discrimination and prediction error. (A) Cumulative/dynamic AUCs at 1, 2, and 3 years for the Tumor DL, VAT DL, preoperative Tumor+VAT, and postoperative multimodal models in the training cohort and two external test cohorts; error bars indicate bootstrap 95% confidence intervals. (B) IPCW Brier-score curves from 1 to 36 months. Survival probabilities were derived from the training-cohort Breslow baseline hazard and locked linear predictors without external-cohort recalibration.

Figure S12. Sensitivity analysis of censoring-adjusted survival decision-curve analysis. IPCW-adjusted net benefit is shown across threshold probabilities of 1%-50% for 1-, 2-, and 3-year recurrence prediction in the training cohort and two external test cohorts. The shaded region denotes the prespecified primary range of 5%-30%. Curves compare the postoperative multimodal model, clinicopathological-only model, pTNM stage model, treat-all strategy, and treat-none strategy.

Figure S13. Variable-selection and model-diagnostic sensitivity analyses. (A, B) Harrell's C-index and 2-year cumulative/dynamic AUC of the original univariable-screened postoperative multimodal Cox model and the all-candidate ridge-penalised Cox model in the training cohort and two external test cohorts; points show estimates and error bars show 1,000-bootstrap 95% confidence intervals. (C) Agreement between the original and ridge-model risk scores. (D) Variable-specific scaled Schoenfeld residual tests for the complete candidate model; the dashed line denotes P = 0.05.

Figure S14. Adjuvant-therapy sensitivity and subgroup analyses. (A) Adjusted associations between the standardised final risk score and recurrence-free survival according to adjuvant-treatment status and pTNM stage. Points show hazard ratios per 1-SD increase and error bars show 95% confidence intervals; interaction P values were derived from pooled continuous interaction models. (B) Absolute standardised mean differences before and after propensity-score overlap weighting for documented postoperative adjuvant therapy. The dashed line denotes an absolute standardised mean difference of 0.10.

Figure S15. Ablation analysis of imaging and clinical model components. (A) Harrell's C-index and (B) 2-year cumulative/dynamic AUC are shown for seven prespecified component combinations in the training cohort and two external test cohorts. The Tumor+VAT model contains only preoperative CT-derived scores, whereas Tumor+VAT+Clinical is the postoperative multimodal model incorporating the seven-variable clinicopathological component. Points indicate estimates and error bars indicate bootstrap 95% confidence intervals.

Figure S16. Training trajectories and checkpoint selection for the original development split. Training Cox loss and training/internal-validation C-indices are shown by epoch for the Tumor DL and VAT DL branches. Dashed vertical lines indicate the checkpoints used to generate the locked scores. These trajectories are presented for transparency and do not establish absence of overfitting.
